# Supplementary material for: FIFA football nurse – A task sharing approach in sports and exercise medicine practice in grassroots women’s football in low- and middle- income settings. A study protocol for a cluster randomised controlled trial
Source: PLoS One. 2023 Sep 21;18(9):e0278428. doi: 10.1371/journal.pone.0278428 (PMC10513307; doi:10.1371/journal.pone.0278428)
Supplement: S1 Protocol — (PDF) [file pone.0278428.s002.pdf]

UNIVERSITY OF MALAWI - COLLEGE OF MEDICINE RESEARCH and ETHICS COMMITTEE (COMREC) CHECKLIST TO ACCOMPANY NEW RESEARCH PROPOSALS SUBMITTED TO THE COMMITTEE

When you submit a research proposal for the Committee to approve, first read the document entitled ***COMREC Elements Of Review*** *attached to this check list***.** *Make sure that your proposal is in the format outlined in the document.*

Before sending or giving the proposal to the Committee, complete the following check-list by ticking each item you have included. Do not submit the proposal *unless you can tick all the boxes, or provide a reason for the absence of any item. Attach the completed check-list to the front of your submission. Provide evidence of payment of US150 processing fee.*

TITLE OF PROPOSAL: Football nurse: A community based, task sharing approach to improve best sports and exercise medicine practice in women's football in Malawi.
**Name of Principal Investigator:** Dr. Enock Chisati
**Name of Sponsor and amount of sponsorship:** Fédération Internationale de Football Association (FIFA), $198 872 (MWK 167 052 480)

Submit all documents in one pdf file of not more than 5MB by email to comrec@medcol.mw (if the file size is more than 5MB, then please zip the file and submit it as a compressed zipped file).

The **single** pdf file should include the following information in the following order:

01	Completed copy of this checklist as stated above		Yes☒or No☐
02	Covering letter of introduction from Investigator		Yes☒or No☐
03	The study protocol which should include the following:-		
	Study Title		Yes☒or No☐
	List of Investigators and institution(s) involved		Yes☒or No☐
	Executive Summary		Yes☒or No☐
	Background/Introduction		Yes☒or No☐
	Rational/justification		Yes☒or No☐
	Objectives of the study: Main objective and Specific Objectives		Yes☒or No☐
	Methods:		
		Type of study - place of study	Yes☒or No☐
		Study population	Yes☒or No☐
		Study period Sample size	Yes☒or No☐
		Data collection procedures	Yes☒or No☐

Approved by College of Medicine
11-Apr-2022
(COMREC) Research and Ethics Committee

TABLE OF CONTENTS

Executive summary	8
Introduction	11
Rationale	15
Study objectives	16
Methods	16
Study type and place	16
Study population	17
Sample size	18
Data collection	19
Data management and analysis	20
Study period	22
Dissemination of results	23
Ethical considerations	23
Possible Constraints	24
Budget and justification	25
References	29
Appendices	31

Approved by
College of Medicine
11-Apr-2022

(COMREC)
Research and Ethics Committee

EXECUTIVE SUMMARY

Research title: Football nurse: A community- based task sharing approach to improve best sports and exercise medicine practice in women's football in Malawi

Study type: This will be a randomised control trial involving women's football teams in the Women's Football League in Malawi.

Problem statement: Football (soccer) is a very popular sport in Africa and player numbers continue to rise at all levels of the sport. This increase in participation is also directly associated with an increase in football related injuries. While participation in football and injuries arising thereof are increasing, there are not enough sports and exercise medicine (SEM) personnel (physiotherapists and doctors) to attend to these football players, which results in an enormous treatment gap in sports injuries and compromises their safe participation in football. This lack of medical care is even more pronounced in women's teams as women and girls tend to bear the brunt in any environment of scarcity.

Low income settings such as Malawi may not currently have enough medical doctors and/or physiotherapists (PT); however, they has relatively higher numbers of other healthcare workers; namely, nurses. While access to a medical doctor or PT may be aspirational for most citizens within the low income settings, nurses provide healthcare from grassroots/community level and sometimes healthcare facilities are exclusively staffed by nurses. Perhaps, instead of having an SEM strategy that is heavily reliant on doctors and PTs as in high income settings, we ought to adapt current global standards to fit the human resources profile of low income settings such as in Malawi and include the largest group of healthcare providers by numbers, nurses.

Actively including nurses into SEM practice in low-income settings may provide an effective, affordable, and sustainable solution to bridge the treatment gap that women football players currently face and allow safer participation in football activities for all, at all levels.

Broad objective

To evaluate the influence of trained nurses as first responders on sports injury related outcomes in women's football teams.

Approved by
College of Medicine
11-Apr-2022

(COMREC)
Research and Ethics Committee

Specific objectives

1. Determine current sports medicine practices in women's football teams in Malawi's Women's Football League.

2. Determine baseline knowledge, attitudes, and behaviours of football stakeholders (players, coaches, team management) in women's football towards injury prevention programs.

3. Identify football injury risks among women's football players in Malawi's Women's Football league.

4. Determine the incidence and prevalence of injuries and illnesses during one competitive season in Malawi's Women's football league.
5. Assess treatment and referral protocols of injuries and illnesses during one competitive season in Malawi's Women's football league.
6. Compare sports medicine practices; injury prevention behaviours; injury risk parameters; incidence and prevalence of injuries and illnesses in teams with and without a Football Nurse during one competitive season in Malawi's Women's football league.

Methodology: This study will be a randomised control trial with teams in the Women's Football League in Malawi. A total of 24 women's football teams playing in the Malawi women's football league will be randomised to either the intervention group or the control group. A cohort of 12 nurses will receive a seven-day training in basic football medicine using the latest version of the FIFA Football Medicine training manuals; after which they will be attached to a total of 12 women football teams during one competitive season. Their roles within the teams will include basic health screening; assessment, immediate care and referral of injuries; as well as maintaining records of player health. The Football Nurses will be directly supervised by and report to a PT or doctor in their district to whom they will refer serious injuries, refer for investigations, or further management. The selected teams and nurses will be followed up for one competitive season and evaluations will be conducted periodically throughout the season and at the end of the intervention. The teams with Football Nurses will be compared to other teams that will not have Football Nurses. The main variables compared between the teams will include awareness and practice of injury prevention programs, and incidence and prevalence of injuries/illnesses.

Approved by
College of Medicine
11-Apr-2022

(COMREC)
Research and Ethics Committee

Expected findings

The study is expected to generate knowledge, attitudes, and behaviours of football stakeholders (players, coaches, team management) in women's football towards injury prevention programs and establish current sports medicine practices in women's football teams in Malawi's Women's Football League. The study will also establish the incidence and prevalence of injuries and illnesses in Malawi's Women's football athletes. Differences in sports medicine practices; injury prevention behaviours; injury risk parameters; incidence and prevalence of injuries and illnesses in teams with and without a Football Nurse during one competitive season in Malawi's Women's football league will also be identified.

Dissemination of results

The study findings will be disseminated to Football Association of Malawi (FAM); women's football clubs in Malawi, the football governing body (FIFA) and the involved District Hospitals. Findings of the study will also be presented at various local and international research conferences and manuscripts arising thereof will be published in relevant peer reviewed journals. Furthermore; a copy of the final research report will be shared with COMREC and KUHeS library.

Approved by
College of Medicine
11-Apr-2022

(COMREC)
Research and Ethics Committee

INTRODUCTION

Football (soccer) is a very popular sport among women and girls in Africa and the numbers continue to rise at all levels of the sport (FIFA, 2014). This increase in participation is also directly associated with an increase in football related injuries as a result *(Drew & Finch, 2016; Moore, Ranson, & Mathema, 2015; Pfirrmann et al., 2016).* Typical football injuries/adverse events include ligament sprains, muscle strains, or concussions *(Freitag, Kirkwood, & Pollock, 2015; Moore, Ranson & Mathema, 2015).* While the biology may be the same, injuries and illnesses in women's football players in low- and middle- income countries (LMIC) such as in sub-Saharan Africa (SSA) have different socioeconomic implications for management and return to play to those faced by their counterparts in high income countries (HIC). This is because trained medical care is very limited in the former *(Lubega., 2020; Chapweteka, 2014; Owoeye et al., 2013; Killowe & Mkandawire, 2005)* compared to the latter *(Malcolm et al., 2017).*

Recent studies on African sport have shown that more than 50% of athletes do not have medical attendees at training or competition *(Lubega et al., 2020; Chapweteka & Rowe, 2014; Owoeye et al., 2013; Killowe and Mkandawire, 2005).* These findings may be attributed to the shortage of trained sports and exercise medicine (SEM) practitioners such as medical doctors (hereinafter referred to as doctors) and/or physiotherapists (PTs) in SSA. In such an environment of scarcity, it is the women, children and the disabled who bear the brunt of these shortages *(Cheteni et al., 2019)* and SEM is no different: medical care is very limited for women and youth football teams worldwide *(Geertsema et al., 2021)* and more so in Africa as what few SEM practitioners there are, tend to practice in (adult) male teams. Consequently, women's

Approved by College of Medicine 11-Apr-2022 (COMREC) Research and Ethics Committee

football players are at the periphery of health services provision in football *(Okholm Kryger et al., 2021),* which compromises their safe participation in the sport *(Tonino & Bollier, 2004; Almquist et al., 2008).* SEM is a relatively new discipline in Africa; therefore, PTs and doctors that may specialise in SEM are fewer *(Chapweteka & Rowe, 2014; Killowe and Mkandawire, 2005)*. Hence, while participation in football and injuries arising thereof are increasing, there are not enough SEM specialists to attend to these football players, which results in an enormous treatment gap in sports injuries. Consequently, 70% of injured athletes do not receive medical treatment or specialist referral following injury on the field of play *(Lubega et al., 2020).*

The seemingly obvious solution to this shortage of trained SEM professionals and subsequent treatment gap in women's football is to train more professionals. However, therein lies another challenge: there are currently not enough institutions that train doctors and/or PTs on the continent to meet the basic health needs of their populations, let alone 'spare' for sport ***(Table 1)***. The current medical best practice in football requires to have at least a doctor, PT and conditioning coach at the pitch side during training and matches *(Casa et al., 2012; Dvorak et al., 2013; Almquist et al., 2008).* This best practice model is based on personnel availability in HIC ***(Table 1).*** Therefore, it may seem impossible to achieve in most teams in LMIC. As a result, most football teams in Africa, especially women's teams, have to do without medical care at training or practice except perhaps at national team level.

Approved by
College of Medicine
11-Apr-2022

(COMREC)
Research and Ethics Committee

Table 1: The number of institutions that train medical doctors and physiotherapists in three selected low-income African countries (Zimbabwe, Uganda, and Malawi) compared to those trained in a high-income country (Australia).

Country	Population (million)	Doctor training institutions (approx. number of graduates per year)	PT training institutions (approx. number of graduates per year)
Zimbabwe	14.7	3 (200)[a]	1 (25)[a]
Uganda	44.3	6 (400)[b]	2 (20)[b]
Malawi	18.6	1 (80)[c]	1 (25)[c]
Australia	25.7	22 (3600)	24 (2500)[d]

[a]Chibhabha, 2021; [b]Lubega, 2021; [c]Phiri, 2021; [d]Kemp, 2021

While SSA may not currently have enough doctors and PTs, SSA also tends to have relatively higher numbers of other health workers; namely, nurses *(Rispel & Bruce, 2014)*. For example, Zimbabwe has 0.24 PTs; 1.6 doctors and 7.2 nurses to 10 000 citizens *(WHO, 2021)* and close to 80% of all healthcare workers in South Africa are nurses *(Rispel and Bruce, 2014).* Consequently, the bedrock of health systems in SSA is the nurse. This heavy reliance on nurses as the main healthcare providers is reflected in the number of nursing schools and graduates in SSA. Zimbabwe and Uganda have 27 *(Nurses Council of Zimbabwe, 2021)* and 50 institutions *(Lubega, 2021; personal communication),* respectively, that train nurses while Malawi graduates approximately 2000 nurses annually *(Phiri, 2021; personal communication).*

In the pyramidal referral system used in many LMIC, doctors are available from secondary health care (e.g. district hospital) and PTs are often available from tertiary health care (e.g. provincial hospital) while nurses are available from the community,

Approved by College of Medicine 11-Apr-2022 (COMREC) Research and Ethics Committee

and primary care levels and throughout the referral pyramid *(Rispel and Bruce, 2014; Sanders et al., 1998).* Hence, while access to a medical doctor or PT may be aspirational for most citizens of SSA, discretionary even, nurses are available to provide healthcare from grassroots level and sometimes healthcare facilities are exclusively staffed by nurses *(Chibanda et al., 2011; Rispel and Bruce, 2014).* A mirror strategy of having nurses available to provide health care from the community and primary care levels with doctors and PTs available at higher levels may be used in football medicine to ensure that health care services are provided for all participants at all levels of the game, especially at grassroots.

Perhaps, instead of having an SEM strategy that is heavily reliant on doctors and PTs as in HIC, we ought to adapt current global standards to fit the human resources profile of SSA and include the largest group of healthcare providers by numbers, nurses. In general medical practice, nurses currently conduct screening, diagnosis and management of medical conditions as well as referring to other healthcare workers as necessary and are an integral part of healthcare delivery in most LMIC *(Chibanda et al., 2011; Rispel and Bruce, 2014).* Similarly, they should be a key component to delivering health care services in football especially at grassroots levels and for underserved football players. Including nurses in the SEM practice agenda may help alleviate the shortage of pitch side medical care in female football teams. Additionally, as nurses are predominantly women *(Rajacich et al., 2013),* their inclusion into SEM practice will also help increase representation of African women SEM practitioners as they are still underrepresented in the profession *(Zondi & Austin, 2021).*

Therefore, the aim of this project is to develop a community and strength based, task sharing approach (Football Nurse) to actively recruit and train nurses as pitch side responders in grassroots women's football. Similar task sharing approaches have

Approved by
College of Medicine
11-Apr-2022
(COMREC)
Research and Ethics Committee

been used successfully in other sporting codes *(World Rugby, 2021)* and in LMIC to manage the diagnosis, treatment, and referral of other health conditions where there is a shortage of specialists *(Chibanda et al., 2011).*

Rationale

Globally, women and girls in sports are confronted with the lack of women friendly sports facilities, equipment, and support personnel. While these barriers to safe participation of women in sport are widely acknowledged, there is a dearth of follow up interventions to specifically address these barriers. In particular, despite an increase in participation in football, most women's football teams in Africa do not have medical care at training or during competition *(Lubega et al., 2020).* This is likely because there are not enough doctors and/or PTs to service these teams. The problem with the status quo is that this inadequate access to sports medical services increases the risk of injury, worsening of injury once incurred, or reinjury *(Owoeye et al., 2013; Almquist et al., 2008; Tonino and Bollier., 2004).* Hence, it is imperative that this gap be filled to ensure safe participation of women players in the game. Nurses are the largest number of healthcare workers by numbers and are the backbone of most health systems in LMIC. Therefore, actively including nurses into SEM practice in these settings may provide an effective, affordable, and sustainable solution to bridge the treatment gap that African women football players currently face and allow safer participation in football activities for all, at all levels. The goal of the Football Nurse project will be to use a task sharing approach to train nurses to provide on field assessment and management of sports injuries in underserved communities such as grassroots women's football. This will ensure that the SEM needs of women football players are met from grassroots level.

Approved by
College of Medicine
11-Apr-2022

(COMREC)
Research and Ethics Committee

STUDY OBJECTIVES

Broad objective

To evaluate the influence of trained nurses as first responders to sports injury related outcomes in women's football teams.

Specific objectives

1. To determine current sports medicine practices in women's football teams in Malawi's Women's Football League.
2. To determine baseline knowledge, attitudes, and behaviours of football stakeholders (players, coaches, team management) in women's football towards injury prevention programs.
3. To identify injury risk among women's football players in Malawi's Women's Football league.
4. To determine the incidence and prevalence of injuries and illnesses during one competitive season in Malawi's Women's football league.
5. To assess treatment and referral protocols of injuries and illnesses during one competitive season in Malawi's Women's football league.
6. To compare sports medicine practices; injury prevention behaviours; injury risk parameters; incidence and prevalence of injuries and illnesses in teams with and without a Football Nurse during one competitive season in Malawi's Women's football league.

METHODS

Study type and place

This will be a parallel randomised controlled pilot study involving all 24 football teams in the Malawi Women's Football League. The 24 teams are located in the following districts: Mzuzu, Mzimba, Lilongwe, Dowa, Kasungu, Blamtyre, Zomba and Chikwawa. The 24 football teams will be randomised to either an intervention group

Approved by
College of Medicine
11-Apr-2022
(COMREC)
Research and Ethics Committee

(IG) or control group (CG). The IG will comprise supervised Football Nurses offering SEM care while the CG will continue SEM practice as is.

To allocate teams to either IG (12 teams) or CG (12 teams), a random sequence of numbers will be generated from the computer using the RANDBETWEEN function in Microsoft Excel, 2016. Groups will then be allocated to teams in sequence using numbered opaque envelopes containing the group allocations. The generation of the number sequence and allocation of the envelopes to the teams will be done by an independent individual who will not be involved in data collection and evaluation of the outcomes.

Study population

The study will involve 24 women's football teams in the women's football league in Malawi. A total of six (6) physiotherapists and/or medical doctors will be recruited from the district hospitals in the district where the women's teams are based. They will serve as supervisors for the Football Nurses during the project. These physiotherapists and doctors will undergo training of Football Medicine practice using the freely and publicly available FIFA Medical Diploma. This will allow for uniformity of football medicine practice and supervision among the participants during the project. After the training of the supervisors, a cohort of 12 nurses will be recruited from health facilities near the women football teams' clubs and training grounds. The 24 women's football teams have been chosen because they are the only teams playing in the only women's football league in Malawi. The nurses will be trained by the investigators using adapted versions of the FIFA Emergency Medicine Manuals over a 7-day period. Each nurse will be allocated to a team whose club base and training grounds are in their district.

Approved by
College of Medicine
11-Apr-2022

(COMREC)
Research and Ethics Committee

Inclusion criteria

- Women's football clubs in the Women's League in Malawi registered with the Football Association of Malawi (FAM).
- Nurses registered with the Nurses and Midwives Council of Malawi with at least 3 years post graduate experience.
- Physiotherapists with a recognised university qualification in physiotherapy (HPT), registered with the Medical council of Malawi and at least 3 years post graduate practice experience.
- Medical doctors with a recognised university qualification (MBChB) registered with the Medical and Dental Association of Malawi and at least 3 years post graduate practice experience.
- Willingness to and actual completion of the online FIFA Medical Diploma over a maximum period of 3-months prior to the commencement of the project.

Exclusion criteria

- Women's football clubs in the Women's League in Malawi that already have medical personnel attached to their team.
- For potential supervisors, less than 3 years post qualification experience; incomplete FIFA Medical Diploma.
- For potential Football Nurses, less than 3 years of post-qualification experience.
- For potential Football Nurses and supervisors, current attachment to a Women's Football club in Malawi's Women's League.

Sample size

The sample size is based on the registered number of women's football teams in the Malawi women's football league. There are a total of 24 female football teams playing

Approved by
College of Medicine
11-Apr-2022
(COMREC)
Research and Ethics Committee

in the Malawi women's league. The study will aim to recruit all the 24 teams, 12 will be in the IG and 12 will be in CG. Twelve (12) nurses will be recruited and one each will be attached to the intervention teams. Six PTs and/or doctors based in the district hospitals where the teams are located will also be recruited to each supervise the Football Nurses from two teams.

Data collection

Prior to the commencement of the intervention and the official start of the season (during pre-season), a survey will be sent out to the women's football teams to determine their current knowledge, attitudes, and behaviours towards injury prevention programs (Appendix B, Section C) and current medical practice (Appendix B, Section D) using tools adapted from previous studies (Bakare et al., 2021; Geertsema et al., 2020; Lubega et al., 2020)

Following this, and prior to the beginning of the season, a cohort of 12 nurses will receive seven day training in basic football medicine using the adapted versions of the latest FIFA Football Medicine training manuals. The main content of the training manuals is listed in (Appendix B, Section Q). The training of nurses will be run by the investigators and will cover didactic, interactive, and practical lectures on common football injuries and medical emergencies with emphasis on recognition, assessment, diagnosis, and immediate care of on field injuries as well as specific considerations for the female football player. The training will also cover the referral pathways of serious cases such as head/ spinal injuries, and fractures.

After training, at the beginning of the season, the nurses will be attached to 12 female football teams in the IG during one competitive season. The CG will continue their current SEM practice as is. The trained nurses will be directly supervised by and report

Approved by
College of Medicine
11-Apr-2022
(COMREC)
Research and Ethics Committee

to a PT or doctor in their district to whom they will refer serious injuries, refer for investigations, or further management. The roles of the nurses within the teams will include menstrual (Appendix B, Section E), basic health (Appendix B, Section F), injury risk assessment (Appendix B, Sections G and H), nutritional (Appendix B, Section J), and mental health assessment (Appendix B, Section K); on field assessment, immediate care and referral of injuries; as well as maintaining records of injuries (Appendix B, Section L), illnesses (Appendix B, Section M), medication use (Appendix B, Section N) and football exposure (Appendix B, Section P) using standardised forms as previously used in African women's football (Mkumbuzi et al., 2021) .

Sections A, B, C1 and C2 of Appendix B will be completed by all participants (PTs, doctors, nurses, players, coaches); Section D will be completed by the researcher for every football team; Sections E to K will be completed by Football Nurses (IG) and Researcher (CG); Sections L to P will be completed by Football Nurses (IG) and Researcher (CG). Sections A to K will be completed; (1) at the beginning of the season/intervention; (2) halfway through the season/ intervention; and (3) at the end of the season/intervention. Sections L to P are to be completed at the end of each training session and/or match throughout the season.

The results from all these will be compared between IG and CG. For teams in the CG, data will be collected by a research assistant who will contact a designated (by the team) member of the management/coaching staff who is responsible for the welfare of the players.

Data management and analysis

Paper copy questionnaires will be stored in locked cabinets in secure building at the Principal Investigator's institution. These will be de-identified and coded immediately

Approved by
College of Medicine
11-Apr-2022
(COMREC)
Research and Ethics Committee

after data collection and only the principal investigator will have access to the master copies with both the names and the codes. Data will be entered into Microsoft Excel sheets and will be stored on a password protected computer database with at least two factor authentication and backed up on a secure cloud based platforms. Data will only be accessed by authorized study personnel and only for research purposes. Each participant will be assigned a unique identification number; the only source of person-identifiable information will only be on the consent forms, which will be in the sole possession of the principal investigator.

The tablet based platforms used to collect and store team data by the Football Nurses and supervisors will be password protected and will require at least two factor authentication to allow access. Each Football Nurse will have access to the complete data set of only their team and each supervisor will have access to the data sets of the two teams under their supervision. The investigators will have access to all datasets. All player health data will be treated as private and confidential and will not be shared with anyone other those providing them with medical care without their verbal assent and written consent.

Data will be analysed using the IBM Statistical Package for Social Sciences (SPSS) version 21. Descriptive statistics using mean and standard deviation (SD) will be used to characterise continuous demographic variables. Student t-tests for continuous variables and chi-square tests for categorical variables will be used to analyse associations and differences before and after the intervention and between the IG and CG. All statistical tests will be two - sided and p values of < 0.05 will be considered statistically significant.

Approved by
College of Medicine
11-Apr-2022

(COMREC)
Research and Ethics Committee

Study period

Activity	Dec 2021- Feb 2022	Jan- Feb 2022	Mar-Apr 2022	April- Nov 2022	Aug 2022-Feb 2023	Mar- Apr 2023
• Proposal writing & development of Football Nurse training materials<br>• Seeking permission from DHOs						
• Ethical review process						
• Recruitment and training of supervisors, football nurses and research assistant						
• Data collection- Football Nurse intervention						
• Data Analyses						
• Manuscript and report writing.<br>• Dissemination of findings						

Approved by
College of Medicine
11-Apr-2022

(COMREC)
Research and Ethics Committee

DISSEMINATION OF RESULTS

The study findings will be disseminated to FAM; women's football clubs in Malawi, the football governing body (FIFA) and the involved District Hospitals. Findings of the study will also be presented at various local and international research conferences and manuscripts arising thereof will be published in relevant peer reviewed journals. Furthermore; a copy of the final research report will be shared with COMREC and KUHeS library.

ETHICAL CONSIDERATIONS

The study proposal will be submitted to the institutional review board (COMREC) for review and ethical approval. Permission was sought and obtained from the Football Association of Malawi (FAM) (Appendix C1), the District Health Officers (Appendix C2) and the District Sports Officiers (Appendix C3) before conducting the research study. The study will be conducted in accordance with the protocol and GCP guidelines. Before they can be a part of the study, the investigators will explain the objectives of the study to the physiotherapists, doctors and nurses; football teams, their women players, and their coaching teams. They will only then be eligible to participate after they have provided verbal and written consent (Appendix A). In the event of a participant being flagged as being at risk of self-harm or harm to others in the mental health assessment, the Football Nurses and their supervisors will be obliged to provide brief intervention within the scope of their practice, following which they will refer the participant for further management by the relevant healthcare providers. Should a medical condition be detected in the physical assessments, a similar approach will be taken in the management of the player. This study will be voluntary and participants will be informed that they have a right to participate or to withdraw from the study at

Approved by
College of Medicine
11-Apr-2022
(COMREC)
Research and Ethics Committee

any time, without prejudice. Data collected from the study will be stored in a password protected database and computer and that will only be accessible to the researchers. Study participants will be assured of their confidentiality. The names of the participants will only be on the consent form and in all other instances, they will be referred to by their assigned unique code.

POSSIBLE CONSTRAINTS

The women's football season may be interrupted by the ongoing COVID-19 pandemic as the footballing season may be extended or suspended. To overcome this, we will work with FAM and focus on data collection during the period when COVID cases are on the decrease while the season is in progress. We will also follow all COVID-19 preventative measures as stipulated by the Government of Malawi.

Approved by
College of Medicine
11-Apr-2022

(COMREC)
Research and Ethics Committee

RESEARCH BUDGET AND JUSTIFICATION

Item	Unit cost (USD)	Quantity required	Justification	Total cost (USD)	Total cost (MK)
**PHASE I- PRELIMINARY WORK**					
Directly incurred costs					
Ethics review fees	500	1	Application for ethical clearance and review for a new research protocol	500	420 000
KUHeS overhead fees	10%	1	KuHES ethical requirement, 10% of total budget	15 164	12 737 760
Phase 1 costs subtotal				***15 664***	***13 157 760***
**PHASE 2- DEVELOPMENT OF CLOUD BASED PLATFORM/TECHNOLOGICAL REQUIREMENTS**					
Directly incurred costs					
- Cloud based tech platform	5 500	1	- Development and maintenance costs of minimum viable product cloud- based platform to compile and store clinical data	5 500	4 620 000
- Cloud services monthly costs	100	12	- Monthly charges by hosting platform to host our data for the duration of the project	1 200	1 008 000
- Maintenance costs of the system	730	12	- Monthly costs of developers to maintain the cloud-based system x 12 months	8 760	7 358 400
- Tablets/Media Pads	200	24	- For Football nurses and supervisors to record injury and training data	4 800	4 032 000
- Project laptops	730	3	- One each for each investigator for research purposes, training, presentations and to ensure a physical back-up of all project-related information	2 190	1 839 600
Phase 2 costs subtotal				***22 450***	***18 858 000***
**PHASE 3A- SUPERVISOR TRAINING WORKSHOP**					

Approved by
College of Medicine
11-Apr-2022
(COMREC)
Research and Ethics Committee

Travel costs					
- Flights for trainers	750	2	-1 X CPT-LLW- CPT (NSM); 1 X DUR-LLW-DUR (PCZ)	1 500	1 260 000
- Local travel	300	1	-Travel to and from the airport, local trips per rising need	300	252 000
Directly incurred costs					
- Accommodation	150	15	- Accommodation and meals, per person per night [(6 rooms sharing x $150/night x 3 days) + (3 rooms x $150/night x 3 days)] at a conference venue in Lilongwe for 3 days and 3 nights	4 050	3 402 000
- Conference venue	100	3	- Conference costs for a venue of less than 30 people for 3 days	300	252 000
- Stationery	190	-	- Cost of stationery for use in the delivery of the workshop	190	159 600
- Internet and Wi-Fi costs	100	3	- Daily internet costs	300	252 000
-Consumables	100	-	- Disposable consumables (e.g. cotton swabs, gloves) for training purposes	100	84 000
Staff costs					
- Trainees	30	12	- Per diem allowances for 12 trainees x 3 days*	1 080	907 200
- Trainers	200	3	- Per diem allowances for 3 trainers x 3 days**	1 800	1 512 000
Phase 3A costs subtotal				***9 620***	***3 166 800***
**PHASE 3B- CLUB RANDOMISATION EVENT**					
Directly incurred costs					
- Refreshments for FAM representatives	100	-	- Refreshments for FAM representatives who will physically attend the randomisation event	100	84 000
- Internet and Wi-Fi costs	200	-	- Internet and communication costs to enable other representatives to attend virtually	200	168 000
Phase 3B costs subtotal				***300***	***252 000***
**PHASE 4- FOOTBALL NURSE TRAINING WORKSHOP**					

Approved by
College of Medicine
11-Apr-2022
(COMREC)
Research and Ethics Committee

Travel costs					
- Flights for trainers	750	2	- 1 X CPT-LLW- CPT (NSM); 1 X DUR-LLW-DUR (SEM	1 500	1 260 000
- Local travel	300	1	Physician)	300	252 000
Directly incurred costs			- Travel to and from the airport, local trips per rising need		
- Accommodation	150	27		15 750	13 230 000
			- Accommodation and meal costs per person per night [(12		
			rooms sharing x $150/night x 7 days) + 3 rooms x		
- Conference venue	100	7	$150/night x 7 days)] at a conference venue in Lilongwe for	700	588 000
- Stationery	350	-	7 days and 7 nights	350	294 000
- Internet and Wi-Fi costs	100	7	- Conference costs for a venue of less than 30 people for 7	700	588 000
- Consumables	300	-	days	300	252 000
Staff costs			- Cost of stationery for use in the delivery of the workshop		
- Trainees	30	24	- Daily internet costs	5 040	4 233 600
- Trainers	200	3	- Disposable consumables (e.g. cotton swabs, gloves) for	4 200	3 528 000
			training purposes		
Phase 4 costs subtotal			- Per diem allowances for 24 trainees x 7 days*	***28 840***	***24 225 600***
			- Per diem allowances for 3 trainers x 7 days**		
**PHASE 5- INTERVENTION**					
Directly incurred costs					
- Internet and communication costs	360	18	- Internet and communication costs to enable communication between Football Nurses, supervisors and research team; uploading of clinical data to cloud system [$40/month x 9 months x 18 participants (12 nurses + 6 supervisors)].	6 480	5 443 200
Staff costs					
- Football Nurses	900	12		10 800	9 072 000
			- Remuneration for Football Nurses for the duration of the program [$100/month x 9 months x 12 nurses]		
- Supervisors	900	6		5 400	4 536 000
Phase 5 costs subtotal			- Remuneration for supervisors for the duration of the program [$100/month x 9 months x 6 PTs/doctors]	***22 680***	***19 051 200***
**PHASE 6- EVALUATION**					
Directly incurred costs					
- Statistics software (STATA; GraphPad Prism; SPSS)	1 040	1	License for 5+ individual licences for use in the statistical analysis of the data.	1 040	873 600

Approved by
College of Medicine
11-Apr-2022
(COMREC)
Research and Ethics Committee

Phase 6 costs subtotal				***1 040***	***873 600***
**OVERALL PROJECT COSTS**					
Directly incurred costs					
- Printer	150	1	- For general office use for the duration of the project	150	126 000
- Stationery	50	9	- Monthly cost of stationery for the duration of the intervention	450	378 000
- General office supplies	50	9		450	378 000
Indirect costs			- For general office use for the duration of the intervention		
- Article processing fees	4 400	3		13 200	11 088 000
- Facilities costs	500	12	- For open access publication of manuscripts arising from this project.	6 000	5 040 000
- Institutional grant management fees	10%	1	- Monthly institutional overheads for use of KuHES office space by the research assistant during the project.	15 164	12 737 760
			- Institutional fees charged at 10% of the total to ensure proper governance of the grant funds		
Staff costs					
- Research assistant	4 800	1		4 800	4 032 000
- Investigators	14 300	3	- To assist in the overall research activities, coordinate capturing and collation of data at the football teams [$5/hr x 4 hr/day x 20 day/month x 12 months]***	42 900	36 036 000
			- Estimated at 2hr/day (25% effort) dedicated to the project (57 000/12 x 12 x 0.25). Each investigator offers subject specific expertise to the project****.	***83 114***	***69 815 760***
Contingency funds	10%	1	Funds for as yet unforeseen circumstances that may arise during the conduct of the project (10%) of subtotal	15 164	12 737 760
**GRAND TOTAL**				**198 872**	**167 052 480**

*based on remuneration rates for local workshops for Malawian institutions; **current remuneration rates per diem FIFA (2019), ***based on prevailing rates for research assistants with no PhD; ****Based on prevailing remuneration for Senior lecturer (USD57 000 p.a) in the sub Saharan region.

Approved by
College of Medicine
11-Apr-2022
(COMREC)
Research and Ethics Committee

REFERENCES

Almquist, J., et al. (2008). Summary Statement: Appropriate Medical Care for the Secondary School-Aged Athlete. *Journal of Athletic Training*, *43*(5), 538–540. www.journalofathletictraining.org

Casa, D., et al (2012). The Inter-Association Task Force for Preventing Sudden Death in Collegiate Conditioning Sessions: Best Practices Recommendations. *Journal of Athletic Training*, 47(4):477–480

Chapweteka, I. (2014). The current state of injury related care for Malawi super league football players. *Masters Thesis*, *University of the Western Cape*.

Chibanda, D., Mesu P., Kajawu L., Cowan F., Araya R., & Abas M.A. (2011). Problem-solving therapy for depression and common mental disorders in Zimbabwe: piloting a task-shifting primary. *BMC Public Health*, *11*(828), 1–10.

Drew MK, Cook J, Finch CF. (2016). Sports-related workload and injury risk: simply knowing the risks will not prevent injuries. *British Journal Sports Medicine*, 50:13 06–08.

Dvorak J, et al. (2013). The FIFA medical emergency bag and FIFA 11 steps to prevent sudden cardiac death: setting a global standard and promoting consistent football field emergency care. *British Journal Sports Medicine, published online* August 12.

FIFA. (2014). FIFA women's football survey. *FIFA, Zurich Switzerland*.

Freitag, A., Kirkwood, G., Scharer, S., Ofori-Asenso, R., & Pollock, A. M. (2015). Systematic review of rugby injuries in children and adolescents under 21 years. *British Journal of Sports Medicine,* 49(8), 511–519.

Geertsema, C., Geertsema, L., Farooq, A., Harøy, J., Oester, C., Weber, A., & Bahr, R. (2021). Injury prevention knowledge , beliefs and strategies in elite female footballers at the FIFA Women's World Cup France 2019. *BrIitish Journal of Sports Medicine*, *0*, 1–6. https://doi.org/10.1136/bjsports-2020-103131

Killowe, C., & Mkandawire, N. . (2005). Knowledge, attitude, and skills regarding sports medicine among football players and team doctors in the football super league in Malawi. *Malawi Medical Journal*, *17*(1), 9–11.

Malcolm, D., Scott-Bell, A., & Waddington, I. (2017). The provision of medical care in English professional football: An update. *Journal of Science and Medicine in Sport*, *20*(12), 1053–1056. https://doi.org/10.1016/j.jsams.2017.05.004

Medical Deans- Australia and New Zealand https://medicaldeans.org.au/data/?md_year=2019&data_type=Graduates&country=AU&students=total&preview=

Moore, I. S., Ranson, C., & Mathema, P. (2015). Injury Risk in International Rugby Union: Three-Year Injury Surveillance of the Welsh National Team. *Orthopaedic Journal of Sports Medicine,* 3(7), 2325967115596194.

Approved by
College of Medicine
11-Apr-2022
(COMREC)
Research and Ethics Committee

Nurses Council of Zimbabwe (2021). Training Institutions. Accessed from http://www.nursescouncil.co.zw/ncz-training-institutions/ 18/06/2021

Okholm Kryger, K., Wang, A., Mehta, R., Impellizzeri, F. M., Massey, A., & McCall, A. (2021). Research on women's football: a scoping review. *Science and Medicine in Football*, *00*(00), 1–10. https://doi.org/10.1080/24733938.2020.1868560

Owoeye, O. B. A., Akinbo, S. R. A., Olawale, O. A., Tella, B. A., & Ibeabuchi, N. M. (2013). Injury prevention in football: Knowledge and behaviour of players and availability of medical care in a Nigerian youth football league. *South African Journal of Sports Medicine*, *25*(3), 77. https://doi.org/10.7196/sajsm.471

Pfirrmann, D., Herbst, M., Ingelfinger, P., Simon, P., & Tug, S. (2016). Analysis of Injury Incidences in Male Professional Adult and Elite Youth Soccer Players: A Systematic Review. *Journal of Athletic Training*, 51(5), 410–424

Rajacich, D., Kane, D., Williston, C., & Cameron, S. (2013). If They Do Call You a Nurse, It Is Always a "Male Nurse": Experiences of Men in the Nursing Profession. *Nursing Forum*, *48*(1), 71–80. https://doi.org/10.1111/nuf.12008

Rispel, L., & Bruce, J. (2014). A profession in peril? Revitalising nursing in South Africa. *South African Health Review*, *1*, 117–125.

Sanders, D, et al., (1998). Zimbabwe's hospital referral system: does it work? *Health Policy Plan, 13*(4), 359-70. doi: 10.1093/heapol/13.4.359. PMID: 10346028.

Tonino, P. M., & Bollier, M. J. (2004). Medical Supervision of High School Football in Chicago: Does Inadequate Staffing Compromise Healthcare? *Physician and Sportsmedicine*, *32*(2), 37–40. https://doi.org/10.3810/psm.2004.02.123

WHO- Global Health Alliance (2021). Zimbabwe. Accessed from https://www.who.int/workforcealliance/countries/zwe/en/#:~:text=Currently%2C%20there%20are%201.6%20physicians,Health%20information%20sheet%2C%202010). 03/05/2021

World Physiotherapy (2021). Member Organisation- Zimbabwe Physiotherapy Association. Accessed from https://world.physio/membership/zimbabwe . 03/05/2021

Zondi, P. C., & Austin, A. V. (2021). A question of colour: Systemic racism in sports and exercise medicine. *British Journal of Sports Medicine*, *55*(10), 526–527. https://doi.org/10.1136/bjsports-2020-103351

.

Approved by
College of Medicine
11-Apr-2022

(COMREC)
Research and Ethics Committee

APPENDICES

Approved by
College of Medicine
11-Apr-2022
(COMREC)
Research and Ethics Committee

Appendix A

Participant information sheet and consent form

FOOTBALL NURSE: A COMMUNITY BASED, TASK SHARING APPROACH TO IMPROVE BEST MEDICAL PRACTICE IN GRASSROOTS FEMALE FOOTBALL IN MALAWI.

Dear Volunteer,

Thank you for agreeing to participate in this study to be conducted by researchers from the Department of Rehabilitation Sciences at the Kamuzu University of Health Sciences.

Why are we doing this study?

Football is a very popular sport in Africa and player numbers continue to rise at all levels of the sport. This increase in participation is also directly associated with an increase in football related injuries. While participation in football and injuries arising thereof are increasing, especially in females, there are not enough sports and exercise medicine (SEM) personnel (physiotherapists and doctors) to attend to these football players, which results in an enormous treatment gap in sports injuries and compromises their safe participation in football. This lack of medical care is even more pronounced in female teams.

Africa may not currently have enough medical doctors and/or physiotherapists (PT); however, it has relatively higher numbers of other healthcare workers; namely, nurses. Nurses provide healthcare from grassroots/community level and sometimes healthcare facilities are exclusively staffed by nurses. Perhaps, instead of having a football medicine strategy that is heavily reliant on doctors and PTs as in high income countries, we should adapt to include the largest group of healthcare providers by numbers, nurses.

What are the aims of this study?

The aims of this study are:

1. To identify the current state of knowledge of injury prevention programs and management of players with injuries or illnesses in women's football teams;
2. To determine the current medical needs of female players in women's football teams;
3. To determine the effects of training nurses as first responders to sports injury management in women's football;
4. To compare injury related outcomes following Football Nurse intervention with usual treatment in women's football teams.

Approved by
College of Medicine
11-Apr-2022
(COMREC)
Research and Ethics Committee

Who can take part in this study?

- **Football clubs**- If you are a women's/girls' football club in Malawi's Super League and do not have a medical professional employed by, on contract, or on retainer with your team,

or

- **Physiotherapists/Medical Doctors**- If you have an interest in sports medicine and have at least 3 years post-graduation experience,

or

- **Nurses**- If you have an interest in sports medicine and have at least 3 years post-graduation experience,

or

- **Football players**- If you are a woman or girl playing football in the Malawi Super League

or

- **Football coaches/managers**- If you coach/manage women and girls playing football in the Malawi Super League.

You cannot take part in this study if:

(i) you are currently under training as a physiotherapist, medical doctor or nurse,
(ii) you have less than 3 years post-graduate experience,
(iii) your team already has medical personnel,
(iv) you are men's or boys' football club,
(v) you are not a football club.

What will happen if you do decide to take part in this study?

Physiotherapists/ medical doctors: You will be recruited as supervisors in this study. During the study, you will be required to complete the FIFA Medical Diploma within a 3-month period. You will then be randomised to be in the control or intervention group. During the conduct of the study, you will provide support and act as the first referral support for the Football Nurses.

Nurses: You will receive a training course on common football injuries and medical emergencies emphasising on recognition, assessment, diagnosis, and immediate care of on field injuries, and considerations for the female football player. The training will also cover the referral pathways of serious cases such as head/ spinal injuries, and fractures. You will then be attached to your respective teams for the duration of the Women's Football season (April to December). During your attachment with the teams, you will attend training sessions and matches and have the following objectives:

1. Basic screening for physical activity (football) readiness.

Approved by
College of Medicine
11-Apr-2022
(COMREC)
Research and Ethics Committee

2. Promote injury prevention practices.
3. Provide primary and secondary assessment of on field injuries.
4. Provide immediate treatment of on field injuries.
5. Refer serious injuries for investigation or further management (including rehabilitation).
6. Follow up on injured players and ensure return to play protocols are observed.
7. Compile and maintain records of player health, training load, injury risk factors, injuries/illnesses surveillance in the team using the Football Nurse Medical Notes platform.

You will be supervised by PTs/ doctors within your district. These supervisors will not necessarily be present at actual training sessions or matches but will have access to any electronic notes on players' injuries/illnesses. When absent from the training field, they will have telephonic access to you should you require immediate assistance at training/ a match. Supervision will be for critical cases such as head/spinal injuries, fractures, medical emergencies or referral for investigations or further management.

For teams and PTs/Drs in the control group, after their training sessions or matches, the researchers will request details from the team manager or coach on any injuries, how and by whom they were managed and whether they were referred for further management.

Football teams: You will attend a randomisation event where we will randomly allocate your team to either the control or intervention group. If you are in the intervention group, during the study, you will provide the Football Nurse access to the women's football team's training sessions and matches throughout the season. If you are in the control group, our researchers will contact you after every training session to ask you questions on who was injured, how and by whom they were managed and whether they were referred for further management.

Football players/coaches: At the beginning of the season, you will be requested to complete questionnaires that will ask for information such as your age, training load, injury history, diet, and injury prevention behaviours. This will take you about 30 minutes to complete. You will then be requested to complete these questionnaires halfway through the season and again at the end of the season. During the rest of the season, we will request information from the nurse attached to your team or your coach/ manager about any injuries or illnesses you get or any medications they might give you during the season. This will not require you to do anything.

What are the risks and discomforts of this study?

We do not foresee any major risks. The completion of questionnaires is not associated with any risk. Questionnaire and other clinical data (paper and electronic) will be kept confidential and secure and will not be made available to any party other than the research team without your consent.

What are the benefits of taking part in this study?

Football teams: If you are in the intervention group, your team will have access to a trained healthcare professional for the duration of the season. If your team is in the control group, you

Approved by
College of Medicine
11-Apr-2022
(COMREC)
Research and Ethics Committee

will receive general feedback at the end of the study describing the feasibility and impact of having a Football Nurse attached to women's football teams. This information will help us to improve medical care in women's football.

Physiotherapists/ Medical doctors: You will be remunerated $100 per calendar month for your expertise and supervisory roles during the conduct of the study.

Nurses: You will be remunerated $100 per calendar month for your services during your attachment to the teams

Football players/coaches: If you are in the intervention group, your team will have access to a trained healthcare professional for the duration of the season. If your team is in the control group, you will receive general feedback at the end of the study describing the feasibility and impact of having a Football Nurse attached to women's football teams. This information will help us to improve medical care in women's football.

What are the ethical considerations?

The Kamuzu University of Health Sciences Research Ethics Committee (contact information below) has approved this study. This study will be performed in accordance with the principles of the Declaration of Helsinki (2013, Fortaleza, Brazil), International Conference on Harmonisation and Malawi Good Clinical Practice (GCP) guidelines and the laws of the Republic of Malawi. The study will be covered by the no-fault insurance policy of the Kamuzu University of Health Sciences. You will not be included in the study unless you have signed a consent form, after the investigator has provided substantial verbal and written explanation of the study, including risk factors. You will be informed that your participation in the study is entirely voluntary and that you have the right to withdraw from the study at any time without stating a reason. The investigator may also withdraw you from the study at any time. All the information collected during the trial will be stored in a computer database in a secure facility, will be kept confidential and will only be used for scientific purposes. Your anonymity will be ensured should the data be published**.** The data collected during the study may be used for future research studies following consultation and permission being granted to do so by the Human Research Ethics Committee of the Kamuzu University of Health Sciences.

What if something goes wrong?

The Kamuzu University of Health Sciences has insurance cover for the event that research-related injury or harm results from your participation in the trial. The insurer will pay all reasonable medical expenses in accordance with the Malawi Good Clinical Practice Guidelines, based on the Association of the British Pharmaceutical Industry Guidelines (ABPI) in the event of an injury or side effect resulting directly from your participation in the trial. You will not be required to prove fault on the part of the University.

The University **will not be liable** for any loss, injuries and/or harm that you may sustain where the loss is caused by:

Approved by
College of Medicine
11-Apr-2022
(COMREC)
Research and Ethics Committee

- The use of unauthorised medicine or substances during the study,
- Any injury that results from you not following the protocol requirements or the instructions that the study doctor may give you,
- Any injury that arises from inadequate action or lack of action to deal adequately with a side effect or reaction to the study medication,
- An injury that results from negligence on your part.

By agreeing to participate in this study, you do not give up your right to claim compensation for injury where you can prove negligence, in separate litigation. In particular, your right to pursue such a claim in a Malawian court in terms of Malawian law must be ensured. Note, however, that you will usually be requested to accept that payment made by the University under the Malawi GCP guideline 4.11 is in full settlement of the claim relating to the medical expenses.

An injury is considered trial-related if, and to the extent that, it is caused by study activities. You must notify the study doctor immediately of any side effects and/or injuries during the trial, whether they are research-related or other related complications.

KUHeS reserves the right not to provide compensation if, and to the extent that, your injury came about because you chose not to follow the instructions that you were given while you were taking part in the study. Your right in law to claim compensation for injury where you prove negligence is not affected. Copies of these guidelines are available on request.

Who do I speak to (or contact) if I have any questions about the study?

Should you have any ethical concerns or questions about the study, please contact:

The Chairperson, COMREC Secretariat, Kamuzu University of Health Sciences (KUHeS), Private Bag 360, Chichiri, Blantyre 3

Tel no. 01871911 (ext. 334)

Mob no.088118993

Email: comrec@medcol.mw.

Should you have any queries directly related to the study itself, please contact any of the investigators:

Dr. Enock Chisati PhD, MSc<br>+265 888 168 284<br>echisati@medcol.mw	Dr. Nonhlanhla Mkumbuzi PhD, PT<br>+27 79 391 6969<br>nonhlanhlamkumbuzi@ntombisport.com

Approved by
College of Medicine
11-Apr-2022

(COMREC)
Research and Ethics Committee

CONSENT TO PARTICIPATE IN STUDY

I, the undersigned, have been fully informed about Kamuzu University of Health Sciences' study entitled '**Football nurse: a community based, task sharing approach to improve best medical practice in grassroots female football in low- and middle- income settings'** to be conducted by researchers from the Department of Physiotherapy.

Football teams

- I agree to have my football team participate in the study.
- I agree to provide the Football Nurses with access to the team's training sessions and matches.

Physiotherapists/Medical Doctors

- I agree to complete the FIFA Medical Diploma in the allotted time.
- I agree to supervise Football Nurses and to be the first point of referral following injury, as the need arises.
- I agree to attend and participate in the 7 day training course in basic football medicine.

Football Nurses

- I agree to attend and participate in the 7 day training course in basic football medicine.
- I agree to perform the tasks for the football teams as explained by the researchers.

Coaches/managers/Football players

- I agree to complete questionnaires on training load, injury history, diet, and injury prevention behaviours.
- I agree to have information on injuries or illnesses that I sustain during the season to be recorded by the football nurse or team manager/ coach.

All participants

- I agree to adhere to all Covid-19 regulations as mandated by the Kamuzu University of Health Sciences' and the Republic of Malawi

I have been fully informed about the risks inherent to participation in this trial. I have had the opportunity to ask questions about the study and had them answered to my satisfaction. I understand that all the information collected during the study will be treated confidentially, will only be used for scientific research purposes and that my name and personal particulars will not be released under any circumstances.

I have been informed that I will be free to withdraw from the study at any time if I so wish without explanation. I will be free to ask any questions about the procedures and results of the study. I understand that I will receive, where applicable, feedback pertaining to the general results of the study once the entire study has been completed.

Approved by
College of Medicine
11-Apr-2022

(COMREC)
Research and Ethics Committee

I agree to participate in the study.

Participant:

______________________	______________________	______________________
Full name	Date	Signature or thumb[illegible]

Investigator:

______________________	______________________	______________________
Full name	Date	Signature

Witness:

______________________	______________________	______________________
Full name	Date	Signature

Approved by
College of Medicine
11-Apr-2022
(COMREC)
Research and Ethics Committee

Appendix A

Uthenga kwa otenga nawo mbali mukafukufuku

NAMWINO WA ZAMPIRA: KUGAWANA NTCHITO POTHANDIZA KUPITITSA PATSOGOLO KASAMALIDWE KA OVULALA MU MPIRA WA MIYENDO WA NTCHEMBERE MABYE KU MALAWI

Okondedwa,

Zikomo kamba kovomera kuti mutenge nawo mbali mu kafukufuku amene akupangidwa ndi anthu aku nthambi yazolimbitsa mafupa ku sukulu ya ukachenjede ya Kamuzu.

Kodi ndi chifukwa chayano tikupanga kafukufuku ameneyi?

Mpira wa miyendo ndi masewero omwe ndi otchuka mu Africa ndipo chiwerengero cha anthu osewera masewerowa chikuchuluka mmagawo onse. Kuchuluka kwa chiwerengeroku ndikomwenso kukupangitsa kukwera kwa chiwerengero cha anthu ovula ku masewero a mpira wa miyendo. Pamene chiwerengero cha masewerowa komanso kuvulala chikukwera, makamaka kwa anthu amayi, kulibe anthu azachipatala odziwa bwino za mankhwala a masewero (madokotala a mafizo komanso a mankhwala). Omwe angathandize amayi omwe akupanga masewero ampira wa miyendo. Izi zapangitsa kuti pakhale kusowekera kwakukulu kwa thandiza lomwe anthuwa akavulala kumasewerowa amalandira ndipo izi zimasokoneza amayiwa kupitiliza masewero ampira. Kusowekera kwa chithandizo cha chipatalaku ndikokwera kwambiri ku ma timu a ntchembere mbaye osewera mpira wa miyendo.

Pakadali pano ku Africa kulibe ma madototo amafizo komanso amankhwala okwanira, koma chiwerengero cha anamwino ndichokwererapo. Anamwinowa omwe ndiochulukirapo kulekana ndi madokotala a mafizo komanso a mankhwala amapezepa muzipatala zazikulu komanso zazing'ono zomwe zimapezeka madera ochuluka a kumidzi. Mwina, m'malo modalira madokotala a mafizo komanso amankhwala omwe ndi ochepa komanso sapezeka mumadera a kumidzi pothandiza ovula a masewera ampira, ndibwino kuyesa kugwiritsa ncthito anamwino omwe ndi ochuluka komanso amapezeka mmadera onse kuphatikizapo akumidzi.

Kodi zolinga za kafukufukuyu ndi ziti?

Zolinga za kafukufukuyu ndi izi:

1. Kuzindikira momwe zinthu ziliri pakadali pano zamapulogalamu opewera kuvulala ndi kasamalidwe ka osewera ovulala kapena matenda m'magulu ampira wamiyendo wa ntchemberembaye;
2. Kudziwa chithandizo chachipatala chomwe chikufumika kwa osewera mpira wa miyendo wa ntchemberembaye;

Approved by
College of Medicine
11-Apr-2022
(COMREC)
Research and Ethics Committee

3. Kudziwa zotsatira za kuphunzitsa anamwino monga oyamba kuthandiza pakuvulala kwa osewera mu mpira wa miyendo wa ntchemberembaye;
4. Kupeza ngati njira yogwiritsa ntchito namwino wa Mpira ingasinthe zotsatira za thandizo lomwe osewera mpira wamiyendo watchemberembaye amalandira poyerekeza ndi thandizo lachizolowezi lomwe osewerawa amapeza.

Ndani yemwe angachite nawo kafukufukuyu?

- **Matimu ampira**- Ngati muli mutimu ya azimayi ntchemberembaye yomwe imasewera mu Super League yaku Malawi ndipo mulibe akatswiri azachipatala omwe amalembedwa ntchito, mwa mgwirizano, kapena osunganso ndi gulu lanu,

Kapena

- **Dotolo wa mafizo/ Dotolo wa mankhwala**- Ngati muli ndi chidwi ndi zamasewero ndipo mwakhala mukugwira ntchitoyi kwa zaka osachepera zitatu mutamaliza maphunziro,

Kapena

- **Namwino**- Ngati muli ndi chidwi ndi zamasewero ndipo mwakhala mukugwira ntchito yanu kwa zaka zosachepera zitatu mutamaliza maphunziro,

Kapena

- **Osewera mpira**- Ngati ndinu mzimayi kapena msungwana yemwe akusewera mpira mu Super League ya Malawi,

Kapena

- **Mphunzitsi wa mpira / Manejala wa ampira**- Ngati mumaphunzitsa / kuyang'anira mpira wa miyendo wa ntchemberembaye mu Super League ya Malawi.

Simungachite nawo kafukufukuyu ngati:

(i) Pakadalipano mukadali ophunzira wa udotolo wa mafizo, mankhwala kapena unamwino;
(ii) Muli ndi zaka zochepera zitatu mutamaliza maphunziro;
(iii) Timu yanu ili kale ndi azachipatala;
(iv) Ndinu timu ya mpira wa miyendo ya amuna;
(v) Timu yanu siyampira wa miyendo.

Chingachitike ndi chiyani mutasankha kutenga nawo mbali mu kafukufukuyu?

Dotolo wa mafizo /mankhwala: Mudzatenganawo mbali ngati oyang'anira mukafukufukuyi, mudzafunika kumaliza maphunziro a FIFA Medical diploma yomwe ndi ya miyezi itatu. Mukatero mudzasinthidwa kuti mukhale mu gulu loyang'anira. Munthawi yakafukufukuyu, mudzakhala mukuthandizira a namwino azampirawa.

Approved by College of Medicine
11-Apr-2022
(COMREC) Research and Ethics Committee

Anamwino: Mudzalandira maphunziro pa zovulala za mumpira komanso zadzidzidzi zamankhwala zomwe ndi kuwunika, komanso kusamalira anthu omwe avulala mu mpira., komanso malingaliro a wosewera wamkazi. Maphunzirowa adzafotokozanso njira zosamalila munthu amene wavulala kwambiri monga kuvulala pamutu /msana, ndi kuthyoka kwa mafupa .Mukatero mudzatumizidwa ku matimu osiyanasiyana munthawi yomwe ligi ya mpira wa miyendo wa ntchemberembaye imaseweredwa. Munthawi yakuphunzira ntchitoyi ndi matimuwa, mukuyenera mukamakakhala nawo munthawi yokonzekera komanso munthawi yamaserewo ndipo mukuyenera mukadziwe izi:

1. Kuwunika koyambirira kwakukonzekera masewera olimbitsa thupi (mpira).
2. Kulimbikitsa njira zopewera kuvulala.
3. Kupeleka ndondomeko zofufuzira kavulalidwe kwa pagilaundi.
4. Kupereka chithandizo mwansanga kwa anthu omwe avulala mu nthawi yamasewero
5. kutumizani onse ovulala kwambiri ku chipatala kuti akalandile chithandizo .
6. Kuchitani kalondolondo wa osewera omwe anavulala ndikuonetsetsani kuti ndondeomeko zowayenereza kubwelelanso mu bwalo lazamasewro zikutsatidwa .
7. Kupangani komanso kusunga mbiri yazaumoyo wa wosewera, chiopsezo chakuvulala kwa mtundu uliwonse mu timu yanu pogwilitsa ntchito nsanja ya Football Nurse Medical Notes.

Mudzayang'aniridwa ndi dotolo wa mafizo wamudera lanu . Okuyang'aniraniwa sadzakhalapo nthawi yamaphunziro anu kapena nthawi yomwe masewro akuchitika koma adzakhala ndi Ufulu owona zonse zomwe mwachita zokhudza kuvulala kapena kudwala kwa osewera. Pomwe iwo sakupezeka kumabwalo azamasewero padzakhala njira zalamya kuti mudztha kulumikizana nawo ngati mungadzafune thandizo lina lililonse nthawi yazokonzekera kapena ku yamasewero. Kukuyang'anirani kudzachitikanso kwambiri ngayti pali kuvulala kodetsa nkhawa ngati kuvulala kwa mutu, msana, kuthyoka kwa mafupa kapena kufunikira kuwonedwa kuchipatala kapena ngati osewera watumizidwa kuchipatala kapena kufunikila koonedwanso mwapaderadera.

Kwa matimu ndi madotolo a mafizo komanso amankhwala mmagulu osayang'aniridwa, pakutha kwa zokonzekera kapena masewero anthu omwe akupanga kafukufukuyi adzafunsa kupatsidwa tsatanetsatane kuchokera kwa oyang'anira timu kapena mphunzitsi wa timu ngati pali ovulala, komanso mmene anthuwa anathandizidwila komanso owathandizirayo komanso ngati anthuwa anatumizidwa kuchipatala kuti akaonedwe mwapadera.

Matimu a mpira: mudzapita ku mkumano umene udzasankhe matimu kuti akhale mu gulu la matimu oyang'aniridwa kapena osayang'aniridwa. Ngati mutakhale mugulu loyang'aniridwa, munthawi ya kafukufukuyi , mukuyenera kuvomera anamwino adzamaserawa kukhala nanu munthawi yanu yazokonzekera komanso yamasewero anu onse amu nyengo yonse yampira. Ngati muli mugulu losayang'aniridwa, anthu amene akupanga kafukufuku adzakuimbirani lamya kapena kupeza njira zina zamkumano kuti amve mmene zokonzekera zanu zayendera komanso amene anavulala, mmene anathandizizdwila komanso kuti anathandizidwa ndi ndani komanso ngati anatumizdwa kuchipatala kuti akaonedwe mwapadera.

Osewera mpira komanso aphunzitsi ampira: Kumayambiriro a nyengo yosewera mpira, mudzafunsidwa kuyankha mafunso omwe akufunsa za zakazanu zakubadwa, ngati munavulalapo, madyedwe anu, komanso mmene mmapewera kuvulala. Izi zidakutengerani mphindi makumi atatu kuti muyankhe. Kenako mudzafunsidwanso kuyankha mafunso ena pakatikati pa nyengo ya mpirayi. Munthawi ya nyengo ya mpirayi, tidzafunsa mafunso kwa anamwino a timu yanu, ophunzitsa kapena oyang'anira timu yanu

Approved by
College of Medicine
11-Apr-2022
(COMREC)
Research and Ethics Committee

pankhani za ovulala kapena odwala omwe munali nawo komanso mankhwala omwe munapatsidwa, inu simudzapangapo kalikonse pa izi.

Kodi ndiziti zomwe zingapereke chiopsezo kapena kusamasuka pamene mukutengapo mbali pakafukuffukuyi?

Sitikuonapo zovuta zimene zingabwere ngati mungatenge nawo mbali mu kafukufukuyi. Mafunso onse komanso zachipatala zonse zidzasungwidwa mwa chinsinsi komanso motetezedwa ndipo izi sizidzapelekedwa kwa munthu aliyense kupatula okhawo amene akupanga kafukufukuyi.

Ubwino otenga nawo mbali mu kafukufukuyi ndi uti?

Matimu ampira: Ngati muli mu gulu loyang'aniridwa, timu yanu idzakhala ndi mwayi okhala ndi munthu wazachipatala okuthandizirani mu nyengo yonse ya mpira. Ngati timu yanu ili mugulu losayang'aniridwa mudzalandila ndondomeko zammene zinthu zayendera pamapeto pa kafukufuyu kufotokozera ubwino wokhala ndi namwino wazampira ku matimu ampira wa miyendo a ntchemberembaye. Izi zidzathandizira ubwino wokhala ndi anthu opeleka thandizo lachipatala ku mpira wamiyendo wa amayi.

Dotolo wa mafizo/mankhwala: Mudzalipidwa ndalama zokwana $100 pa mwezi kamba ka upangiri wanu komanso ntchtio yoyang'anira panthawi yakafukufukuyi.

Namwino: Mudzalipidwa ndalama zokwanira $100 pa mwezi paupangiri umene mudzapeleke munthawi imene mukugwila ntchito ku matimu osiyanasiyana.

Osewera mpira komanso aphunzitsi a mpira: Ngati muli mugulu loyang'aniridwa, timu yanu idzakhala ndi mwayi okhala ndi munthu wazachipatala okuthandizirani mu nyengo yonse ya mpira. Ngati timu yanu ili mugulu losayang'aniridwa mudzalandila ndondomeko zammene zinthu zayendera pamapeto pa kafukufuyu kufotokozera ubwino wokhala ndi namwino wazampira ku matimu a mpira wa miyendo wa ntchemberembaye. Izi zidzathandizira ubwino wokhala ndi anthu opeleka thandizo lachipatala ku mpira wamiyendo wa amayi.

Zovomerekeza mu kafukufukuyu ndi ziti?

Chilolezo cha kafukufukuyu chatengedwa ku Committee yoona nkhani zosaphwanya maufulu a otenga nawo mbali mukafukufuku ya Sukulu ya ukachenjede ya Kamuzu University of Health Sciences (KUHeS). Kafukufufkuyi achitika mostsatila ndondomeko zonse zoyenera za 2013 za Helsinki komanso nthambi yaoona kuti anthu zachipatala akupanga zinthu zoyenera komanso malamulo amdziko la Malawi. Kafukufukuyi adzatsatilanso ndondomeko za KUHeS. Simudzaikidwa pa mndandanda opanga nawo kafukufukuyi ngati inu eni ake simunavomeleze kutero pamene amene akupanga kafukufukuyi afotokoza zonse zofunikila. Mudzadziwitsidwanso kuti kutenga mbali kwanu pa kafukufukuyu sikokakamiza koamsno muli ndi ufulu osiya kuyankha mafunso akafukufukuyi pomwe mwafunira komanso mosakakamizidwa. Komanso opanga kafukfufukuyu atha kukuchotsani pa ndondomeko yoyankha nawo mafundso ntawi ina iliyonse. Zonse zomwe zidzatoleledwe nthawi ya kafukufukuyi zidzasungidwa bwino ndiponso mwachinsinsi. Maina anu sadzatchulidwa kwinakuli konse ndipo zonse zidzakhala zachinsinsi ngati zopezedwa pakafukufukuyu zitadzalembedwe mmabukhu. Zopezedwa pa kafukfufukuyu zithanso kudzagwilitsidwa ntchito ku kafufufuku wina yemwe angadzachitike ndipo nthambi yaza kafufkfufuku ku sukulu yaukachenjede ya KUHaS ndiyomwe ingadzapereke chilolezochi.

Approved by
College of Medicine
11-Apr-2022
(COMREC)
Research and Ethics Committee

Chingachitike ndi chani ngati china chake chatalakwika?

Sukulu ya ukachenjede ya KUHeS ili ndi ndondomeko zothandizira anthu omwe angakumane ndi zovuta zina munthawi ya kafukufukuyu. Ndondomekoyi idzalipila zofunika kuchipatala malingana ndi ndondomeko za Malawi Good Clinical practice potengeranso British Pharmacutical Industry Guidelines ngati china chake chitavuta kutsatira kafukufukuyi. Sukulu ya ukachenjedeyi sidzakhudzidwa ndi kutayika kapena kuvulala komwe kungadzakhalepo ngati zinthuzo zachitika kamba ka izi:

- Kugwilitsa ntchito mankhwala osavomelezeka kapena zinthu zina munthawi ya kafukfufukuyu.
- Kuvulala kwa njira iliyonse yomwe idzachitike kamba kakusatsata zoyenelela komanso ndondomeko zimene mutadzapatsidwe mukafukufukuyu.
- Kuvulala kwa njira iliyonse yomwe itadzachitike kamba kosasamala kapena kusatsata ndondomeko zothanilana ndi mavuto ena mukafukfufukuyu.
- Kuvulala kwinakulikonse kochitika kamba ka kunyalanyaza kwanu.

Pamene mwavomereza kutenga nawo mbali mu kafukufuku ameneyu, sizikutantahuza kuti mulibe ufulu ofuna chipepeso ngati mwavulala pokhapokha nagti mwapeleka umboni. Muli ndi ufulu opita ku mabwalo azamilandu kuti malamulo agwile ntchito. Koma mukuyenera kudziwa kuti mudzapemphedwa kulandila ndalama imene sukulu yaukachenjede ya KUHeS yavomereza pansi pa ndondomeko za GCP 4.11 polipila zonse zokhudzana ndi ku chipatala. Mukuyenera kudziwitsa a dotolo amene alipo mukafukufukuyu ndi cholinga choti adziwe ngati pali zovuta zina pamene mukuyesedwa komanso ngati zili zoyambitsidwa ndi kafukufukuyu kapena zinthu zina .

KUHeS siidzapeleka chipepeso chinachilichonse ngati kuvulala kwanu kuli kodza kamba ka kusasamala kwanu posatsatira ndondomeko zonwe munapatsidwa kuti mutsatire pamene mumatenganawo mbali mukafukufukuyu. Koma ufulu wanu opatsidwa chipepeso sukuphwanyidwa ngati mwapeleka umboni okwanira. Mutha kupatsidwa ndondomeko zokhudza izi ngati mwapempha.

Ndingayankhule ndi ndani ngati ndil ndi mafunso okhudza kafukufukuyi?

Ngati muli ndi mafunso kapena zovuta zina zokhudzana ndi kafukufukuyu lembani kalata kwa wapampando potumiza ku adiresi ili pansipa kapena imbani lamya pa manambala omwe ali pansipa:

Wapampando
COMREC Secretariat
Kamuzu University of Health Sciences (KUHeS)
Private Bag 360
Chichiri
Blantyre 3
Lamya: 01871911 (ext. 334) / 088118993
Email: comrec@medcol.mw.

Ngati muli ndi zina zofuna kudziwa zokhudzana ndi kafukufuyu, yankhulani ndi omwe akuchita kafukufukuyu omwe mayina awo ali pansipa;

Approved by
College of Medicine
11-Apr-2022
(COMREC)
Research and Ethics Committee

Dr. Enock Chisati PhD, MSc<br>+265 888 168 284<br>echisati@medcol.mw	Dr. Nonhlanhla Mkumbuzi PhD, PT<br>+27 62 340 8451<br>nsmkumbuzi@gmail.com

Approved by
College of Medicine
11-Apr-2022
(COMREC)
Research and Ethics Committee

KUVOMEREZA KUTENGA NAWO MBALI MUKAFUKUFUKUYI

Ine , otenga nawo mbali , ndafotokozeredwa zonse za kafukufuku yemwe sukulu ya ukachenjede ya KUHeS ikufuna kupanga otchedwa **Namwino wa za mpira: kugawana ntchito pothandiza kupititsa patsogolo kasamalidwe ka ovulala mu mpira wa miyendo wa ntchemberembaye ku Malawi** yemwe adzapangidwe ndi anthu ochita kafukyufuku ochokera ku nthami yazamafizo.

Matimu ampira

- Ndavomereza kuyi timu yanga ipange nawo kafukufukuyu.
- Ndavomereza kupeleka chilolezo kwa anamwino azampira kuti atha kumapezeka nawo ku masewero athu komanso zokonzekera za timuyi.

Dotolo wa mafizo/mankhwala

- Ndavomera kumaliza FIFA medical diploma munthawi yoyenelela.
- Ndavomera kuyang'anira anamwino ampira wa miyendo komanso kukhala munthu oyamba kuthandiza munthu akavulala pomwe pali kufunikila kutero.
- Ndavomereza kupezeka komanso kutenga nawo mbali ku maphunziro amasiku asanu ndi awiri okhudza zamankhwala a zampira.

Anamwino azamasewero a mpira wamiyendo

- Ndavomereza kupezeka komanso kutenga nawo mbali ku maphunziro amasiku asanu ndi awiri okhudza ndi mankhwala a zampira .
- Ndavomera kugwira ntchito zonse ku matimu ampira wamiyendo monga momwe afotokozera omwe akupanga kafukufuyi.

Aphunzitsi ampira /oyang'anira matimu ampira komanso osewera mpira

- Ndavomera kuyankha mafunso onse omwe ndapatsidwa, zokhudzana ndi mbiri yovulala, madyedwe komanso kapewedwe ka kuvulala.
- Ndikuvomera kusunga uthenga okhudza kuvualala kapena kudwala komwe kungandichitikire munyengo ya mpira kuti zilembedwenso ndi namwino waza mpira, mphunzitsi kapena oyang'anira timu.

Otenga nawo mbali onse

- Ndavomera kutsata ndondomeko zonse zopewera matenda a Covid-19 monga momwe ikunenera sukulu ya ukachenjede ya KUHeS komanso dziko la Malawi.

Ndauzidwa mwatsatanetsatane zonse zokhudzana ndi kafukufuku ameneyu. Ndinapatsidwanso mwayi ofunsa mafunso ndipo ndayankhidwa mokhutitsidwa. Ndamvesetsanso kuti zonse zomwe zitatoleledwe mukafufkufukuyu zikhala za chinsinsi ndipo zigwilitsidwa ntchito mwaukadaulo komsno kuti maina satchulidwa kapena kugawidwa ntchito kwina kuli konse.

Ndamvesetsanso kuti ndikhonza kusiya kutenga nawo mbali mukafukuffukuyu popanda kukakamizidwa komanso kunena zifukwa zomwe ndikusiila. Ndili ndi ufulu ofunsa mafunso okhudza ndondomeko

Approved by
College of Medicine
11-Apr-2022
(COMREC)
Research and Ethics Committee

komanso zotsatira za kafukufukuyu. Ndamvesetsanso kuti ndizauzidwa zotsatira paliponse pofunikila pamene kafukufukuyu watha.

Ndavomera kutenga nawo mbali mukafukufukuyu.

Otenga nawo mbali

Dzina	Tsiku	posaina kapena chidindo

Ofufuza

Dzina	Tsiku	posaina

Mboni

Dzina	Tsiku	Posaina

Approved by
College of Medicine
11-Apr-2022
(COMREC)
Research and Ethics Committee

participant code…………

APPENDIX B- DATA COLLECTION QUESTIONNAIRES

INSTRUCTIONS

1. Sections **A, B, C1, C2** are to be completed by all participants (PTs, Drs, nurses, players, coaches, managers).
2. Section **D** to be completed by the **researcher** for **every football team.**
3. Sections **E** to **K** are to be completed by **Football Nurses** (IG) and **Researcher** (CG).
4. Sections **L** to **P** are to be completed by **Football Nurses** (IG) and **Researcher** (CG) throughout the season.
5. Sections **A** to **K** are to be completed (1) at the beginning of the season/intervention, (2) halfway through the season/ intervention, and (3) at the end of the season/intervention.
6. Sections **L** to **P** are to be completed at the end of each training session and/or match.

Approved by
College of Medicine
11-Apr-2022
(COMREC)
Research and Ethics Committee

participant code…………

Section A: DEMOGRAPHIC DATA	
Team<br>District	
What is your date of birth?	
What is your gender?	☐ Male ☐ Female ☐ Other
What is the highest level of education/training you have attained?	☐ Primary school ☐ Secondary school<br>☐ Tertiary- Diploma ☐ Tertiary- Bachelors<br>☐ Tertiary- Masters ☐ Tertiary- Doctorate
What is your position in the football team?	☐ Player ☐ Coach<br>☐ Football nurse ☐ Team/squad physiotherapist<br>☐ Team/squad doctor ☐ Team/squad welfare manager<br>☐ Team/squad strength and conditioning coach<br>☐ Match official (e.g. referee, umpire)<br>☐ Administrator (e.g. Chef de Mission, country/sport representative)<br>☐ Other (specify)……………………………………………………………
When did you start participating in football (year)?	
At what level are you currently involved in your sport?	☐ Amateur ☐ Semi-professional<br>☐ Professional ☐ Other (specify)……………………………………….
If you are a player, what is your coach's gender?	☐ Male ☐ Female ☐ Other
If you are team support personnel (e.g. coach, manager), what is your management's predominant gender? (excluding players)	☐ Male ☐ Female ☐ Other
Section B: INJURY MANAGEMENT PROCEDURES	

Approved by
College of Medicine
11-Apr-2022
(COMREC)
Research and Ethics Committee

participant code…………

1a. Does your team have access to a medical professional?	☐ Yes ☐ No
If the answer above is no, continue to question 2, if yes respond to question 1b.	
1b. If yes, which one (s)?<br>☐ physician (doctor) ☐ physiotherapist<br>☐ nurse ☐ sports scientist<br>☐ Other (specify)……………	
2. How often do you have access to the medical professional?	
Very frequently | *Frequently* | *Occasionally* | *Rarely* | *Never*	
3. At your club, if a player gets injured during training, who treats their injuries?	
1. At your club, if a player gets injured/ill during a match, who treats their injuries	
2. At your club, after a player gets injured/ill who do they go to for further management?	
3. Does your team have medical aid?	☐ Yes ☐ No
4. Do you have personal medical aid?	☐ Yes ☐ No
5. If you answered no to questions 7 or 8 above, who pays for the management of your injuries/illnesses?	
6. Have you been injured in the last 12 months?	☐ Yes ☐ No
7. If you answered yes above, where were you injured?<br>☐ head/face ☐ sternum/ribs/upper back ☐ neck<br>☐ abdomen ☐ shoulder/clavicula ☐ low back/sacrum/pelvis<br>☐ upper arm ☐ hip/groin ☐ forearm<br>☐ thigh ☐ elbow ☐ knee<br>☐ wrist ☐ lower leg/Achilles tendon ☐ hand/finger/thumb<br>☐ ankle ☐ foot/toe ☐ breast	
8. Were your injuries considered serious?	☐ Yes ☐ No
9. Did you receive any treatment after being injured?	☐ Yes ☐ No
10. Who paid for the costs of treatment?	

Approved by
College of Medicine
11-Apr-2022
(COMREC)
Research and Ethics Committee

participant code…………

**Section C1: INJURY PREVENTION BELIEFS** *(adapted from Bakare et al., 2021; Geertsema et al., 2021)*					
	**Strongly agree**	**Agree**	**Don't know**	**Disagree**	**Strongly disagree**
Injury prevention programmes are important in football					
A medical support system is important in achieving goals of injury prevention					
Coach's injury prevention practices is sufficient to achieve injury prevention goals					
Player's knowledge is important in achieving injury prevention goals					
Identifying injury risk factors and modifying them contributes to achieving injury prevention goals					
It is key to work as a team to achieve the goals of injury prevention					
It is more important to use training time to play football than to do injury prevention exercises?					
Injury prevention is directly related to team success					
Compliance to an injury prevention program is important in achieving its set goals					
Coach's beliefs are important in achieving injury prevention goals					
The motivation of the coach affects the players' motivation to do injury prevention exercises					
How do you feel about injury prevention measures?					
The FIFA 11+ injury prevention program in important in achieving injury prevention goals in football					
An implementation guide is important in achieving injury prevention goals					
Beliefs significantly affect injury prevention practices					

Approved by
College of Medicine
11-Apr-2022
(COMREC)
Research and Ethics Committee

participant code…………

Injury prevention practices enhances performance optimisation					
Low muscle strength causes injury					
Poor pitch quality causes injury					
Artificial turf causes injury					
Too much training causes injury					
Too little training causes injury					
Reduced recovery causes injury					
Hard tackles cause injury					
Too many matches cause injury					
Reduced flexibility causes injury					

Approved by
College of Medicine
11-Apr-2022
(COMREC)
Research and Ethics Committee

participant code…………

Section C2: INJURY PREVENTION BEHAVIOURS (*adapted from Bakare et al., 2021; Geertsema et al., 2021*)					
**Do you engage in any football injury prevention?**	☐ Yes ☐ No				
**Which type of injury prevention do you practice?**	**Never**	**Daily**	**Once a week**	**Twice a week**	**Three times a week**
FIFA 11+					
General warm-up					
Sports-specific programme					
Strength training on the pitch					
Strength training before or after football					
Strength training in separate session					
Flexibility on the pitch					
Flexibility before or after football					
Flexibility in separate session					
Sprint training					
Cool-down					
Other (specify)…………………………………………					
**Did you receive any training in the injury prevention practices you engage in?**	☐ Yes		☐ No		
**When do you carry out injury prevention programmes?**	☐ Match days		☐ Training		☐ Both
**For training, when do you carry out injury prevention programmes?**	☐ Self- training		☐ Team training		☐ Both
**Do you think your injury prevention practices you engage in are based on scientific research?**	☐ Yes		☐ No		
**Do you think you will be willing to adopt a different injury prevention strategy from what you already engage in?**	☐ Yes		☐ No		

Approved by
College of Medicine
11-Apr-2022
(COMREC)
Research and Ethics Committee

participant code…………

**Do you think you have adequate knowledge about injury prevention in football?**	☐ Yes	☐ No
**Do you think you effectively apply this knowledge as a football player/coach?**	☐ Yes	☐ No
**Do you think your current injury prevention strategy works?**	☐ Yes	☐ No
**Have you heard about the FIFA 11+ football injury prevention programme?**	☐ Yes	☐ No
**Are you interested in adopting the FIFA 11+ football injury prevention programme?**	☐ Yes	☐ No

Approved by
College of Medicine
11-Apr-2022
(COMREC)
Research and Ethics Committee

participant code……….

Section D1: CURRENT MEDICAL PRACTICE *(Adapted from Lubega et al., 2020)*					
**Q1: In your job, are you required to contribute to a players or athlete's wellbeing?**	☐ Yes ☐ No				
If the answer is **no** move to question **2**, if the answer is **yes,** continue with **1b & c**					
**1b. In your opinion, how important are these factors to a female player's wellbeing?**					
	Very important	*Important*	*Moderately important*	*Of little importance*	*Unimportant*
Doping					
Coaching					
Sports performance					
Injury and illness prevention					
Mental health					
The menstrual cycle					
Nutrition					
**1c. How do you rate your own competency in assisting the player in managing the following categories or circumstances?**					

Approved by
College of Medicine
11-Apr-2022
(COMREC)
Research and Ethics Committee

participant code…………

	Very good	*Good*	*Barely acceptable*	*Poor*	*Very poor*
Doping					
Coaching					
Sports performance					
Injury and illness prevention					
Mental health					
The menstrual cycle					
Nutrition					

**Q2: Do you know the goals and objectives of any of the following organisations? WHO, IOC, FIFA, and WADA**	☐ Yes	☐ No

If the answer is **No** move to question 3, if the answer is **Yes,** continue to **2b & c**

2b. In sourcing information on the wellbeing of players, how important are these international organisations?

	Very important	*Important*	*Moderately important*	*Of little importance*	*Unimportant*
WHO					
IOC					

Approved by College of Medicine
11-Apr-2022
(COMREC) Research and Ethics Committee

FIFA					
WADA					
**2c. In sourcing information on the wellbeing of players or athletes, how often do you use these international organisations' websites?**					
	Very frequently	*Frequently*	*Occasionally*	*Rarely*	*Never*
WHO					
IOC					
FIFA					
WADA					
**Q3: How do you rate your knowledge on the following topics?**					
	Extremely poor	*Below average*	*Average*	*Above average*	*Excellent*
Periodic Health Evaluation (PHE) as part of the players' healthcare program.					
Pre-participation Examination (PPE) is part of healthcare program for players.					
**Q4: Rate the following statement as applicable to you.**					

Approved by
College of Medicine
11-Apr-2022
(COMREC)
Research and Ethics Committee

participant code………..

	Strongly agree	*Agree*	*Undecided*	*Disagree*	*Strongly disagree*
When players undergo periodic health examination (PHE), there is a need to do pre-participation examination also (PPE).					
**Q5: How often do you use the following examples on your players?**					
	Very frequently	*Frequently*	*Occasionally*	*Rarely*	*Never*
I use Periodic Health Evaluation to monitor the players' wellbeing.					
I use Periodic Health Evaluation to educate players about other health risk related behaviours					
**Q6: In your position, are you required to administer basic life support?**				☐ Yes	☐ No
If the answer is **No** move to question 5, if the answer is **Yes** move to section b & c					
**6b. If a player collapses and is unconscious with a suspected foreign body airway obstruction, (choking). In your opinion, it is right to <u>firstly</u>;**					
	Strongly agree	*Agree*	*Undecided*	*Disagree*	*Strongly disagree*
I call the ambulance first and then do CPR					
I call the ambulance while simultaneously giving 1-5 back blows					

Approved by
College of Medicine
11-Apr-2022
(COMREC)
Research and Ethics Committee

I call the ambulance while encouraging a player to cough					
**6c. In your position, how often do you use the following protocols as primary care of a calf muscle strain of player or athlete on the sports court or field?**					
	Very frequently	*Frequently*	*Occasionally*	*Rarely*	*Never*
Danger Respond Airway, Breathing, Circulation (DRABC)					
Airway, Breathing, Circulation, Disability & Exposure (ABCDE)					
Stop, Analyze, Listen, Touch, Active, Passive & Support (SALTAPS)					
Rest, Ice, Compression & Elevation (RICE)					
**Q7: Have you attended or treated an athlete or player with an acute musculoskeletal injury?**				☐ Yes	☐ No
If the answer is No move to question 6, if the answer is **Yes** move to section b & c					
**7b. Which of the following protocols would be appropriate for an injured player?**					
**Contusion**	*Strongly agree*	*Agree*	*Undecided*	*Disagree*	*Strongly disagree*
Danger Respond Airway, Breathing, Circulation (DRABC)					
Airway, Breathing, Circulation, Disability & Exposure (ABCDE)					
Stop, Analyze, Listen, Touch, Active, Passive & Support (SALTAPS)					

Approved by
College of Medicine
11-Apr-2022
(COMREC)
Research and Ethics Committee

participant code…………

Rest, Ice, Compression & Elevation (RICE)					
**Muscle rupture**	*Strongly agree*	*Agree*	*Undecided*	*Disagree*	*Strongly disagree*
Danger Respond Airway, Breathing, Circulation (DRABC)					
Airway, Breathing, Circulation, Disability & Exposure (ABCDE)					
Stop, Analyse, Listen, Touch, Active, Passive & Support (SALTAPS)					
Rest, Ice, Compression & Elevation (RICE)					
**Concussion**	*Strongly agree*	*Agree*	*Undecided*	*Disagree*	*Strongly disagree*
Danger Respond Airway, Breathing, Circulation (DRABC)					
Airway, Breathing, Circulation, Disability & Exposure (ABCDE)					
Stop, Analyse, Listen, Touch, Active, Passive & Support (SALTAPS)					
Rest, Ice, Compression & Elevation (RICE)					
**Ankle fracture**	*Strongly agree*	*Agree*	*Undecided*	*Disagree*	*Strongly disagree*
Danger Respond Airway, Breathing, Circulation (DRABC)					

Approved by
College of Medicine
11-Apr-2022
(COMREC)
Research and Ethics Committee

Airway, Breathing, Circulation, Disability & Exposure (ABCDE)					
Stop, Analyse, Listen, Touch, Active, Passive & Support (SALTAPS)					
Rest, Ice, Compression & Elevation (RICE)					
**7c. Dressings and bandages are used for…?**					
	Strongly agree	*Agree*	*Undecided*	*Disagree*	*Strongly disagree*
Reducing pain					
Reducing internal bleeding					
To help control bleeding					
Prevention of infection					
Make it easier to remove the player after injury					
**Q8: In your position, do you ever refer players to other health service providers?**				☐ Yes	☐ No
If the answer is **No** move to question 9, if the answer is **Yes** move to section b & c					
**8b.Who do you think a player, who has sustained the following injuries during participation in football activities, should be referred to?**					
**Concussion**	*Strongly agree*	*Agree*	*Undecided*	*Disagree*	*Strongly disagree*

Approved by
College of Medicine
11-Apr-2022
(COMREC)
Research and Ethics Committee

Physiotherapist					
Cardiologist					
Orthopaedic surgeon.					
Radiologist for scan or x-ray					
Traditional healer					
General doctor					
Sports physician					
No one					
Other (specify) ………………………..					
**Suspected cardiac condition**	*Strongly agree*	*Agree*	*Undecided*	*Disagree*	*Strongly disagree*
Physiotherapist					
Cardiologist					
Orthopaedic surgeon.					
Radiologist for scan or x-ray					
Traditional healer					

Approved by
College of Medicine
11-Apr-2022
(COMREC)
Research and Ethics Committee

participant code...........

General doctor					
Sports physician					
No one					
Other (specify)					
**Suspected internal injuries**	*Strongly agree*	*Agree*	*Undecided*	*Disagree*	*Strongly disagree*
Physiotherapist					
Cardiologist					
Orthopaedic surgeon.					
Radiologist for scan or x-ray					
Traditional healer					
General doctor					
Sports physician					
No one					
Other (specify)					

Approved by
College of Medicine
11-Apr-2022
(COMREC)
Research and Ethics Committee

participant code……….

**Suspected fracture**	*Strongly agree*	*Agree*	*Undecided*	*Disagree*	*Strongly disagree*
Physiotherapist					
Cardiologist					
Orthopaedic surgeon.					
Radiologist for scan or x-ray					
Traditional healer					
General doctor					
Sports physician					
No one					
Other (specify) …………………………					
**Ligament sprain**	*Strongly agree*	*Agree*	*Undecided*	*Disagree*	*Strongly disagree*
Physiotherapist					
Cardiologist					
Orthopaedic surgeon.					

Approved by
College of Medicine
11-Apr-2022
(COMREC)
Research and Ethics Committee

Radiologist for scan or x-ray					
Traditional healer					
General doctor					
Sports physician					
No one					
Other (specify) …………………………….					
**8c. How often do you refer a player with a suspected concussion to see the following?**					
	Very frequently	*Frequently*	*Occasionally*	*Rarely*	*Never*
Physiotherapist					
Cardiologist					
Orthopaedic surgeon.					
Radiologist for scan or x-ray					
Traditional healer					
General doctor					
Sports physician					

Approved by
College of Medicine
11-Apr-2022
(COMREC)
Research and Ethics Committee

No one					
Other (specify) ………………………..					
**8d. How often do you refer a player with a suspected cardiac condition to see the following?**					
	Very frequently	*Frequently*	*Occasionally*	*Rarely*	*Never*
Physiotherapist					
Cardiologist					
Orthopaedic surgeon.					
Radiologist for scan or x-ray					
Traditional healer					
General doctor					
Sports physician					
No one					
Other (specify) ………………………..					
**8e. How often do you refer a player with a suspected fracture to see the following?**					

Approved by
College of Medicine
11-Apr-2022
(COMREC)
Research and Ethics Committee

participant code…………

	Very frequently	*Frequently*	*Occasionally*	*Rarely*	*Never*
Physiotherapist					
Cardiologist					
Orthopaedic surgeon.					
Radiologist for scan or x-ray					
Traditional healer					
General doctor					
Sports physician					
No one					
Other (specify) …………………………					

8f. How often do you refer a player with a suspected ligament sprain to see the following?

	Very frequently	*Frequently*	*Occasionally*	*Rarely*	*Never*
Physiotherapist					
Cardiologist					

Approved by
College of Medicine
11-Apr-2022
(COMREC)
Research and Ethics Committee

Orthopaedic surgeon.					
Radiologist for scan or x-ray					
Traditional healer					
General doctor					
Sports physician					
No one					
Other (specify) …………………………					

**Q9: In your position, are you required to record and report sports injury circumstances when they occur or when players report such incidence?**	☐ Yes	☐ No

If the answer is **No** move to question 10, if the answer is **Yes** move to section b & c,

9b. In your opinion, which of the following statements are correct?

	Strongly agree	*Agree*	*Undecided*	*Disagree*	*Strongly disagree*
A recurrent injury is an injury of the same type, which occurs after a player's return to full participation from the previous injury					

Approved by
College of Medicine
11-Apr-2022
(COMREC)
Research and Ethics Committee

The severity of an injury is the number of days that have elapsed from the date of injury to the date of the player's return to full participation in team training and availability for match selection.					
A recurrent injury is an injury of the same type and at the same site as an previous injury and which occurs after a player's return to full participation from the previous injury.					
**9c. Which of the following tools do you use often for monitoring and reporting of sports injury circumstances?**					
	Very frequently	*Frequently*	*Occasionally*	*Rarely*	*Never*
SCAT (Standard Concussion Assessment Tool)					
FIFA Injury report forms					
IOC- Injury report form					
GPS (Global Positioning System)					
Daily Analysis of Life demands of Athletes (DALDA					
**Q10: Do you provide treatment to players' injuries after their participation in football activities?**				☐ Yes	☐ No
If the answer is **No** move to question 11, if the answer is **Yes** move to section b & c,					
**10b. In your opinion, would you treat the following cases using surgical means?**					

Approved by
College of Medicine
11-Apr-2022
(COMREC)
Research and Ethics Committee

participant code…………

	Strongly agree	*Agree*	*Undecided*	*Disagree*	*Strongly disagree*
Ankle sprain					
Muscle contusion					
Fractured tibia					
Concussion					
Shin splints					
Anterior cruciate ligament (ACL) rupture					
**10c. Do you provide non-surgical treatment to the following sports injuries?**					
	Very frequently	*Frequently*	*Occasionally*	*Rarely*	*Never*
Ankle sprain					
Muscle contusion					
Fractured tibia					
Concussion					
Shin splints					

Approved by
College of Medicine
11-Apr-2022
(COMREC)
Research and Ethics Committee

participant code…………

Anterior cruciate ligament (ACL) rupture					
**Q11: For your job, have you managed a concussed player or athlete before?**				☐ Yes	☐ No
If the answer is **No,** move to question 12, if the answer is **Yes** move to section b & c					
**11b. Indicate your level of agreement with the following statements:**					
	Strongly agree	*Agree*	*Undecided*	*Disagree*	*Strongly disagree*
A concussed player or athlete is assessed on the side-line, referred to hospital or medical centre, then after, start the return-to-play protocol.					
For suspected concussed player or athlete-on pitch/side-line is assessed, then after, start the return-to-play protocol.					
For a player or athlete diagnosed with concussion, the return-to-play protocol will depend on the time symptoms are resolved.					
During the treatment of a concussed player, assist a player in activities that require concentration and attention until symptoms are absent for a minimum of 24hrs consecutively without medication					

Approved by
College of Medicine
11-Apr-2022
(COMREC)
Research and Ethics Committee

participant code………..

**Q12: For your job, are you required to manage or participate during a player's rehabilitation phase?**	☐ Yes ☐ No
If the answer is **Yes** move to section b & c, if the answer is no, thank you for attending to the above questions.	

12b. The following specialists are important during the rehabilitation phase of a player or athletes with a hamstring injury.

	Strongly agree	*Agree*	*Undecided*	*Disagree*	*Strongly disagree*
Sports Physician					
Physiotherapist					
Strength and conditioning coach					
Psychologist and/or Social worker					
Nutritionist/dietitian					
Surgeon					
Cardiologist					
Occupational therapist					
Sports/technical analyst					
Urologist/gynaecologist					
Nurse					

Approved by
College of Medicine
11-Apr-2022
(COMREC)
Research and Ethics Committee

participant code…………

Chiropractor					
Traditional healer					
Spiritual/faith healer					
Other (specify) ………………………					
**12c. In your situation, which of the following statements are applicable during the rehabilitation phase of an injured player?**					
	Strongly agree	*Agree*	*Undecided*	*Disagree*	*Strongly disagree*
I work with their families and friends					
I respect his/her dignity or rights as other patients					
I monitor & evaluate his/her recovery stages on a daily basis					
I / we allow advice on diet and fluids					
I advise on use of banned substances					
I allow talking to the teammates and visiting friends					
I work with sports physicians					
I / we allow religious leaders or cultural leaders as part of the team during the rehabilitation phase					

Approved by
College of Medicine
11-Apr-2022
(COMREC)
Research and Ethics Committee

participant code…………

I / we evaluate athlete/player's recovery depending on athletes' symptoms resolution					
It is the duty of a physiotherapist to perform periodic health examination and pre-participation examination					
It is the duty of a coach to perform a periodic health evaluation and pre-participation examination					

Approved by
College of Medicine
11-Apr-2022
(COMREC)
Research and Ethics Committee

participant code………..

Section D2: SPORTS FACILITY CHECKLIST *(Adapted from Lubega et al., 2020)*

	None	Poor	Partial	Complete	Notes
**Stadium/playing field management**					
Signposts indicating direction					
Type of playing field/surface (grass/Astroturf)					
Condition of playing surface					
Play dimensions standard (L=105, W= 68: Surface area; L=125, W=80)					
Presence of obstacles on playing surface					
Emergency services (fire warning & detection system, security officer in charge)					
Floodlights					
**Playing quality of football playing surface**					
Ball rebound resilience					
Ball rolling resistance					
Traction					
Hardness of the surface					
Surface evenness					
Water infiltration rate					
FIFA standard field & 5m area space around it					
Field fixtures					
Goal posts & nets white, with grey goal posts, nets and corner flags close to field					
Boundary boards					
Spectator area accessibility for individuals with disability					
**Hygiene and sanitation**					
Toilets					
Change rooms (team, referees, & officials)					
Dustbins					
**Athletes' personal protective equipment**					

Approved by
College of Medicine
11-Apr-2022
(COMREC)
Research and Ethics Committee

participant code………..

Right sized gloves (goalkeepers)					
Right sized footballs					
Shin pads					
Footwear					
Athletes' uniforms (colour and fit)					
**Stadium health care services**					
Availability of medic					
Qualifications of medic					
Medic's awareness of policies concerning sports health care					
Doping control station					
Athlete medical care rooms (couch, oxygen & AED available & audited)					
Ambulance services availability					
Availability of emergency plan					
Injury surveillance reports					
Communication tools on the field (cell phone or walkie-talkies					

Approved by
College of Medicine
11-Apr-2022
(COMREC)
Research and Ethics Committee

participant code………..

Section E: MENSTRUAL HISTORY

At what age did you start menstruating? (years)	
Are you currently using any type of contraception?	☐ Yes ☐ No
If **Yes**, what type of contraception are you using?	☐ Pill ☐ Injection ☐ IUD ☐ Rods<br>☐ Other (Specify)………………………..
If **No**, have you used contraception in the past?	☐ Yes ☐ No
Have you ever been pregnant?	☐ Yes ☐ No
Have you given birth before?	☐ Yes ☐ No
When was your last period *(the first day of your last period)*?	

Generally, how long is your menstrual cycle? *(days from one period to the other)*		How long do you bleed for when menstruating? (days)	

Do you consider your menstrual cycles regular?	☐ Yes ☐ No	
Do you suffer/have you suffered from any menstrual/ hormonal conditions	☐ Yes ☐ No	If **yes**, please select from the list below

☐ Dysmenorrhoea (period pain)
☐ Amenorrhoea (no periods)
☐ Menorrhagia (heavy bleeding)
☐ Endometriosis
☐ Polycystic Ovarian Syndrome (PCOS)
☐ Other (specify)…………………………………………

Do you take any medications during your period?	Yes	No		
If **yes**, what kind of medications do you take?	Painkillers (over the counter)	Painkillers (prescribed)	Herbal preparations	Other (specify)
Do you have access to your preferred sanitary ware?	Yes	No		

If **yes**, what is your preferred choice of sanitary ware? (you can choose more than one)

Sanitary pads	Tampons	Menstrual cups	Cotton wool	Other (specify)

Approved by College of Medicine
11-Apr-2022
(COMREC)
Research and Ethics Committee

participant code…………

Which absorbency level of sanitary ware do you use?			
Light	Regular	Maxi (super)	Maxi (super) plus

Generally, how many times a day do you change your sanitary ware?	
Do you sometimes use double sanitary protection (e.g. pad and tampon)?	☐ Yes ☐ No

If you **do not** have access to your preferred sanitary ware, what do you use when you are menstruating?				
Old clothes-rags	Newspaper	Leaves	Animal dung	Other

Do your periods affect your life in general?	☐ Yes ☐ No
If **yes**, is the effect	☐ Positive ☐ Negative
Do your periods affect your sporting participation?	☐ Yes ☐ No
If **yes**, is the effect	☐ Positive ☐ Negative

Approved by
College of Medicine
11-Apr-2022
(COMREC)
Research and Ethics Committee

participant code...........

Section F: PHYSICAL ACTIVITY READINESS QUESTIONNAIRE

**General medical questions***	Yes	No
Has your doctor ever said that you have a heart condition and that you should only perform physical activity recommended by a doctor?		
Do you feel pain in your chest when you perform physical activity?		
In the past month, have you had chest pain when you were not performing any physical activity?		
Do you lose your balance because of dizziness or do you ever lose consciousness?		
Do you have a bone or joint problem that could be made worse by a change in your physical activity?		
Is your doctor currently prescribing any medication for your blood pressure or for a heart condition?		
Do you know of any other reason why you should not engage in physical activity?		
**Occupational questions**		
What is your current occupation? ____________________		
Does your occupation require extended periods of sitting? ____________________		
Does your occupation require extended periods of repetitive movements? (If yes, please explain.) ____________________ ____________________		
Does your occupation require you to wear shoes with a heel (dress shoes)? ____________________		
**Recreational questions**		
Do you partake in any recreational activities (golf, tennis, skiing, etc.)? (If yes, please explain.)		
Do you have any hobbies (reading, gardening, working on cars, exploring the Internet, etc.)? (If yes, please explain.)		
**Medical questions**		

Approved by
College of Medicine
11-Apr-2022
(COMREC)
Research and Ethics Committee

Have you ever had any pain or injuries (ankle, knee, hip, back, shoulder, etc.)? (If yes, please explain.)<br>____<br>____<br>____		
Have you ever had any surgeries? (If yes, please explain.)<br>____<br>____		
Has a medical doctor ever diagnosed you with a chronic disease, such as coronary heart disease, coronary artery disease, hypertension (high blood pressure), high cholesterol or diabetes? (If yes, please explain.)<br>____<br>____<br>____		
Are you currently taking any medication? (If yes, please list.)<br>____<br>____<br>____		

**If she answered "Yes" to one or more of the above questions, refer to supervisor before engaging in football activities.*

Approved by
College of Medicine
11-Apr-2022
(COMREC)
Research and Ethics Committee

participant code…………

SECTION G: PAST HISTORY OF INJURIES

Please complete this section for each injury. If you have had more than one past injury additional forms will be available.

Have you **ever** in your career suffered from **any injury** (pain, swelling, stiffness) in any body part? Yes ☐ No ☐

If **YES,** please complete the rest of the section below:-

If **NO**, you have completed the questionnaire.

Please tick which **body parts** you have injured recently (past six months).

Also indicate (tick) if your injury was longsatnding pain or an acute injury.

Body region	Side		Injury type		Date of injury
	Left	Right	Longstanding injury	Acute/ short term injury	
Head/face					
Neck					
Chest/breasts/ ribs					
Abdomen (belly)					
Back					
Elbow					
Shoulder					
Wrist					
Finger/ hand					
Hip/ groin					
Thigh					
Knee					
Ankle					
Foot/toe					

Approved by
College of Medicine
11-Apr-2022
(COMREC)
Research and Ethics Committee

participant code…………

SECTION H: INJURY RISK ASSESSMENT			
**LIMB GIRTHS (cm)**			
	First	Second	Third
**Upper limb (Bicep)**			
**Chest**			
**Abdominal (navel)**			
**Mid- thigh**			
**Mid- calf**			
**ANKLE DORSIFLEXION (cm)**			
	First	Second	Third
**RIGHT**			
**LEFT**			
**HAMSTRING FLEXIBILITY (cm)- sit and reach**			
	First	Second	Third
**LUMBAR EXTENSION (cm)**			
	First	Second	Third
**LUMBAR FLEXION (cm)**			
	First	Second	Third

MUSCLE ENDURANCE						
**Push-ups**		**Prone elbow plank (sec)**	**Side plank (L)-sec**	**Side plank (R)-sec**	**Squats**	
30s	*60s*	-			*30s*	*60s*

Approved by
College of Medicine
11-Apr-2022
(COMREC)
Research and Ethics Committee

participant code…………

FUNCTIONAL MOVEMENT SCREENING								
	R	L	R	L	R	L	R	L
Deep overhead squat								
Hurdle step								
In-line lunge								
Active straight leg raise								
Trunk stability push up								
Seated rotary stability								
Shoulder mobility								

Approved by
College of Medicine
11-Apr-2022
(COMREC)
Research and Ethics Committee

participant code…………

Section J: NUTRITIONAL ASSESSMENT- 24 HOUR FOOD RECALL QUESTIONNAIRE

FOOD DESCRIPTION	FOOD SOURCE						TIME		PORTION SIZE		
	Self/home made	Ready to eat	Takeaway/restaurant	Mix	Work/school	Other	PM	AM	How many?	How much?	How thick?

Approved by
College of Medicine
11-Apr-2022
(COMREC)
Research and Ethics Committee

SECTION L: INJURY SURVEILLANCE FORM

1A Date of injury: **1B Date of return to full participation:**

2A Injured body part

☐ Head/face	☐ shoulder/clavicula	☐ hip/groin
☐ Neck/cervical spine	☐ upper arm	☐ thigh
☐ Sternum/ribs/upper back	☐ breast	☐ elbow
☐ knee	☐ Abdomen	☐ forearm
☐ lower leg/Achilles tendon	☐ Low back/sacrum/pelvis	☐ wrist
☐ ankle	☐ Hand/finger/thumb	☐ foot/toe

2B Injured body part ☐ Right ☐ left ☐ not applicable

3 Type of injury

☐ concussion with loss of consciousness ☐ concussion without loss of consciousness ☐ lesion of meniscus or cartilage

☐ haematoma/contusion/bruise ☐ fracture ☐ muscle rupture/strain/tear/cramps

☐ abrasion ☐ laceration ☐ other bone injury ☐ dislocation/subluxation

☐ tendon rupture/injury/bursitis ☐ sprain/ligament injury ☐ nerve injury

☐ dental injury ☐ other injury (specify)…………………………………………………………………………

4 Diagnosis (text or Orchard code):…………………………………………………………………………………

5 Has the player had a **previous injury** of the same type at the same site (i.e. this injury is a recurrence)?

no ☐ yes

If **YES,** specify date of player's return to full participation from previous injury:

6 Was today's injury caused by **overuse** or **trauma?** ☐ Overuse ☐ trauma

7 When did today's injury occur? ☐ Training ☐ Match [☐ 1st half ☐ 2nd half]

8 Was the injury caused by **contact** or **collision?**

☐ No ☐ yes, with another player ☐ Yes, with the ball ☐ Yes, with other object (specify)

9 Did the referee indicate that the action leading to the injury was a **violation of the laws?**

No ☐ yes, free kick/penalty ☐ yes, yellow card ☐ yes, red card

If **YES**, was the referee's sanction against ☐ injured player ☐ opponent

10 Will you conduct further investigations for this injury? ☐ No ☐ Yes (specify)……………………………………

11 Will you refer this injury for further management? ☐ No ☐ Yes (specify)……………………………………

12 Will the player miss training/competition because of the injury? ☐ No ☐ Yes (how many days?)……………

Approved by
College of Medicine
11-Apr-2022
(COMREC)
Research and Ethics Committee

SECTION M: ILLNESS REPORT FORM

Date	Player no.	Illness/Ailment	Cause/aetiology	Treatment administered

Approved by
College of Medicine
11-Apr-2022
(COMREC)
Research and Ethics Committee

SECTION N: MEDICATION USE REPORT FORM

Date	Player no.	Drug class	Dosage	Reason for prescription and dispensing

Approved by
College of Medicine
11-Apr-2022
(COMREC)
Research and Ethics Committee

SECTION P: FOOTBALL EXPOSURE REPORT

Date	Match/ training	Number of players	Duration of match/training session (minutes)	Type of training conducted

Approved by
College of Medicine
11-Apr-2022
(COMREC)
Research and Ethics Committee

Nambala ya wotenga nawo mbali……………….

APPENDIX B
MAFUNSO A MCHICHEWA (MBALI ZOKHAZO ZOMWE ZILI ZOYENERA)

GAWO A: ZOKHUDZANA NDI WOTENGA MBALI PA KAFUKUFUKU	
Boma la team	
Tsiku lobadwa	
Kodi ndinu mwamuna kapena mkazi?	☐ mamuna ☐ mkazi ☐ zina
Maphuzilo munapita nawo patali bwanj?	☐ sukulu ya primary ☐ sukulu ya secondary<br>☐ sukulu ya ukachenjede- Diploma ☐ Sukulu ya ukachenjede- Bachelors<br>☐ Sukulu ya ukachenjede- Masters ☐ Sukulu ya ukachenjede- Doctorate
Udindo wanu ndotani mu team ya mpira yo?	☐ Osewera ☐ Mphunzitsio wa mpira<br>☐ Namwino wa team ya mpira ☐ Dotolo wa mafizo<br>☐ Dotolo wa team ☐ Woyang'anira za umoyo wa onse<br>☐ phunzitsi wa mafizo<br>☐ Oyimbila mpira (e.g. referee, umpire)<br>☐ Oyang'anila (e.g. Chef de Mission, country/sport representative)<br>☐ Zina (longosolani)…………………………………………………
Munayamba liti kumenya mpira wa miyendo? (Chaka)?	
Muli pa mlingo wanji mumasewelawa?	☐ Oyamba kumene ☐ Katswiri<br>☐ Katswiri kwambili ☐ Zina(longosolani)…………………………………
Ngati muli osewela mpira, coach wanu ndiotani?	☐ Mamuna ☐ Mkazi ☐ Zina
Ngati muli othandizila team (e.g. coach, manager), Anthu ambili ndiotani?(kuphatikizila osewera mpira)	☐ Mamuna ☐ Mkazi ☐ Zina
**GAWO B: NDONDOMEKO YOTHANDIZIRA OSEWERA AKAVULALA**	
1a. kodi team yanu imakwanisa kukumana ndi katswiri wa zachipatala?	☐ Eya ☐ Ayi

Approved by
College of Medicine
11-Apr-2022
(COMREC)
Research and Ethics Committee

Nambala ya wotenga nawo mbali……………….

Ngati mwayankha kuti ayi pitani ku funso 2,ngati mwayankha kuti eya pitani ku funso 1b.				
1b. Ngati eya, ndi uti?<br>☐ Dotolo ☐ Physiotherapist (wamafizo)<br>☐ Namwino ☐ sports scientist<br>☐ Zina (longosolani)……………				
2. Mumakumana kangati ndi katswiri wa chipatala?				
Pafupi pafupi kwambili	*Pafupi pafupi*	*Osati kwambili*	*Patali patali*	*Sizichitika*

3. ku club kwanuko ngti osewela wavulala nthawi ya masewera okonzekera, amamuthandiza ndani?	
1. ku club kwanuko ngati osewela wavulala kapena kudwala nthawi yomenya ndi team ina, amamuthandiza ndani?	
2. ku club kwanuko ngati osewela wavulala kapena kudwala, amapita kwa ndani kuti alandile thandizo lina?	
3. Team yanu ili ndi thandizo la zachipatala (medical aid)?	☐ Eya ☐ Ayi
4. Inuyo muli ndi thandizo la zachipatala (medical aid)?	☐ Eya ☐ Ayi
5. Ngati mwayankha kuti ayi pa funso 7 kapena 8 mmwambamu,amalipila ndani kuti ovulala athandizidwe?	
6. Kodi mwavulalako mu miyezi khumi ndi iwiri yadutsayi?	☐ Eya ☐ Ayi
7. Ngati mwayankha kuti Eya mmwambamu,munavulala pati?<br>☐ mutu/nkhope ☐ chifuwa ☐ khosi<br>☐ Mimba ☐ phewa ☐ Chiuno<br>☐ Mkono wa mmwamba ☐ Nyung'unyu ☐ Nkono<br>☐ Ntchafu ☐ gongono ☐ Bondo<br>☐ Polumikizira dzanja ☐ Polumikizira phazi ☐ Dzanja/Chala/Chala chachikulu<br>☐ Chigonkhono ☐ Phazi	
8. Kodi kuvalala kwanuko kunali kwakukulu?	☐ Eya ☐ Ayi
9. Kodi munalandila thandizo lililonse mutavulala?	☐ Eya ☐ Ayi
10. Kodi analipila ndani kuti mulandile thandizolo?	

Approved by
College of Medicine
11-Apr-2022
(COMREC)
Research and Ethics Committee

Nambala ya wotenga nawo mbali………………..

GAWO C1: ZIKHULUPIRIRO ZOKHUDZANA NDI NJIRA ZOPEWERA KUVULALA (zotengedwa kuchokera kwa Bakare et al., 2021; Geertsema et al., 2021)					
	Kugwilizana nazo kwambili	**Kugwilizana nazo**	**Sindikuziwa**	**Sindikugwiliza nazo**	**Ndakana**
Njira zopewela kuvalala mu mpira nzofunika					
akatswiri azachipatala angathandizile kupewa kuvulala					
Goals Njira za a coach nzokwanila kupewa kuvulala					
Nzeru za osewera mpira nzofunika kupewa kuvulala mu mpira					
Identifying injury risk factors and modifying them contributes to achieving injury prevention goals					
nzofunika kugwila nchito ngati team kuti mupewe kuvulala					
Kodi Nzofunika kwambili kugwilitsa nchito nthawi kukonzekera masewelo a mpira a miyendo kusiyana nkumapanga njira zopewela kuvulala mu mpira?					
Injury prevention is direct Njira zopewela kuvulala kumakhuzana kwambili ndi momwe gulu likuchitila bwino					
kutsatila njira zopewela kuvulala nkofunika kukwanilisa zotsatila zomwe njira zo zinapangilidwila					

Approved by
College of Medicine
11-Apr-2022
(COMREC)
Research and Ethics Committee

Nambala ya wotenga nawo mbali………………..

Zikhulupiliro za a coach nzofunika pokwanilitsa zifukwa zomwe mukupangila njira zopewela kuvulala					
Chilimbikitso Cha coach chimakhuzana ndi Chilimbikitso cha osewera mpira kuti apange njira zopewela kuvulala					
Mumamva bwanj pa nkhani ya njira zopewela kuvulala?					
Njira ya FIFA 11+ ndiyofunika kukwanilitsila zifukwa zomwe mukupangila njira zopewela kuvulala					
Njira zokhazikitsidwa nzofunika popewa kuvulala					
Zikhulupiliro zimakhuzana ndi njira zopewela kuvulala					
Njira zopewela kuvulala zimathandiza kuti masewelo akhale apamwamba					
kuchepa mphavu Kwa nyama kumabweletsa kuvulala					
posewelela mpira poyipa pamabweletsa kuvulala					
posewelela pamakono pamabweletsa kuvulala					
kukonzekera masewelo kwambili kumabweletsa kuvulala					
kukonzekera masewelo kochepa kumabweletsa kuvulala					
kusewela Munthu usanachile mokwanila kumabweletsa kuvulala					
kulandana mpira mwamphavu kwambili kumabweletsa kuvulala					

Approved by
College of Medicine
11-Apr-2022
(COMREC)
Research and Ethics Committee

kusewela mpira kwambili kumabweletsa kuvulala					
kufewa Kwa thupi kochepa kumabweletsa kuvulala					

GAWO C2: MAKHALIDWE OPEWA KUVULALA (adapted from Bakare et al., 2021; Geertsema et al., 2021)					
Kodi mumapanga makhalidwe opewa kuvulala mu mpira wa miyendo?	☐ Eya ☐ Ayi				
Kodi ndi khalidwe lake liti lopewa kuvalala mu mpira wa miyendo mumapanga?	**Sitipanga**	**Tsiku lililonse**	**Kamodzi pa sabata**	**Kawiri pa sabata**	**Katatu pa sabata**
FIFA 11+					
General warm-up					
Sports-specific programme					
Strength training on the pitch					
Strength training before or after football					
Strength training in separate session					
Flexibility on the pitch					
Flexibility before or after football					
Flexibility in separate session					
Sprint training					
Cool-down					
Zina (Longosolani)……………………………………………					
Kodi munalandilapo ukadawulo mu njira zopewela kuvulala?	☐ Eya ☐ Ayi				
Kodi mumapanga nthawi zake ziti ukadawulo wopewa kulala wo?	☐ Tsiku lomenya mpira ☐ Kokonzekela kumenya mpira ☐ Zonse				

Approved by College of Medicine
11-Apr-2022
(COMREC) Research and Ethics Committee

Nambala ya wotenga nawo mbali……………….

Mukamakonzekela maseweloKodi mumapanga nthawi zanji ukadawulo wopewa kuvulala?	☐ Kukonzekela ngati team ☐ Pokonzekela ngati team ☐ Zonse
Kodi mukuganiza kuti njira zopewela kuvulalazi zinachokela ku kafukufuku wa science?	☐ Eya ☐ Ayi
Kodi muli okonzeka kuti mutha kuphuzira njira zina zopewela kuvulala?	☐ Eya ☐ Ayi
Kodi mukuwona ngati muli ndi nzeru zokwanila zopewela kuvulala mu mpira wa miyendo?	☐ Eya ☐ Ayi
Kodi mukuwona ngati mumagwilitsa nchito bwino nzeru zo monga inu osewera/coach?	☐ Eya ☐ Ayi
Kodi mukuwona ngatinjira zanu zopewela kuvulala zimagwira?	☐ Eya ☐ Ayi
Kodi munamvapo za njira yopewela kuvulala mu mpira wa miyendo ya FIFA 11+ ?	☐ Eya ☐ Ayi
Kodi muli ndi chidwi chokuti muyambe kugwilitsa nchito njira yopewela kuvalala mu mpira wa miyendo ya FIFA 11?	☐ Eya ☐ Ayi

Approved by
College of Medicine
11-Apr-2022
(COMREC)
Research and Ethics Committee

Nambala ya wotenga nawo mbali……………….

GAWO F: MAFUNSO OKHUDZANA NDI MAJOWAJOWA

Dzina:____ TSIKU:__________________
UTALI: __________cm.   KULEMERA:____________kg.
CHAKA CHOBADWA:___________yrs

Mafunso oyambirira	Eya	Ayi
Kodi muwudzidwapo ndi dotolo kuti muli ndi vuto la ntima ndipo mukuyenela kuchita masewera olimbitsa nthupi ovomelezeka ndi dotolo?		
Kodi mumamva ululu pachifuwa mukamachita masewero olimbitsa nthupi?		
Kodi mwedzi wapitawu, munamvapo ululu pa chifuwa mutakhala osachita masewero olimbitsa nthupi?		
Munayambapo mwafuna kugwa chifukwa chachizugulire kapena munakomokapo?		
Kodi muli ndi vuto la mafupa kapena mavuto a pamalo omwe mafupa amagwirana amene atha kuwonjedzereka Kamba kakusintha momwe mumachitira masewelo olimbitsa nthupi?.		
Kodi mwalembeledwa ndondomeko yamakhwala ndi dotolo wanu okhudza kuthamanga kwa magadzi kapena vuto la ntima?		
Kodi pali chifukwa chilichotse chomwe chingakukanikitseni kutenga nawo mbali pa masewelo olimbitsa nthupi?		
**Mafuso okhudza ntchito**		
Kodi mumagwira ntchito yanji?<br>____		
Kodi ntchito yanu imafunika kukhala pansi nthawi yayitali?<br>____		
Kodi ntchito yanu imanika kuyenda yenda? (ngati eya, chonde fotokozani.)<br>____<br>____		
Kodi ntchito yanu imafunika kuvala nsapato dzadzitali chidendene (dress shoes)?<br>____		
**Mafuso a zamasewera**		
Kodi mumatenga nawo mbali pa masewelo otsangalasa monga (gofu, tenesi, kupaga siki, ena.)? (ngati eya, chonde fotokodzani.)		
Kodi muli ndi chilichose chomwe chimakusangalatsani (Kuwerenga, Ulimi, kugwilira tchito mu galimoto, Kugwiritsa ntchito intaneti, ena.)? (If yes, please explain.)		
**Mafuso azaumoyo**		

Approved by
College of Medicine
11-Apr-2022
(COMREC)
Research and Ethics Committee

Kodi munamvapo ululu kapena kuvulala (chidendene, bondo, nchafu, kumbuyo, phewar, ena.)? (ngati eya, chonde fotokodzani.)<br>____________________<br>____________________<br>____________________		
Kodi munapangidwapo maopaleshoni? (ngati eya, chonde fotokodzani.)<br>____________________<br>____________________		
Kodi munayezedwapo matenda awa, matenda a ntima, nthenda okhudza mitsempha ya ntima, kuthamanga magazi,<br>coronary heart disease, coronary artery disease, hypertension (high blood pressure), kuchuluka kwa kolestero kapena matenda a suga? (ngati eya, chonde fotokodzani.)<br>____________________<br>____________________<br>____________________		
Kodi mukulandira makhwala ena aliwonse? (ngati eya, chonde tchulani.)<br>____________________<br>____________________<br>____________________		

* *Ngati mwayankha kuti "eya" mwalimodzi kapena kuposela limodzi kumafutso omwe ali m'mwambawa, kumanani ndi dokotala musanayambe kutenga nawo mbali pa masewera olimbitsa thupi. Mudziwitseni dokotala wanu mafuso omwe mwayankha kuti "eya". Akatha kukuukanikani achipatala, futsani maganizo kwa dotolo wanu za masewera olimbitsa thupi omwe mukuyenela kutega nawo mbali ogwirizana ndi nyengo yomwe muli.*

Approved by
College of Medicine
11-Apr-2022
(COMREC)
Research and Ethics Committee

Nambala ya wotenga nawo mbali………………..

GAWO J: ZOKHUDZANA NDI ZAKUDYA PA MAOLA MAKUMI AWIRI APITAWA

Zakudya	KAPEZEDWE KA ZAKUDYA						NTHAWI		KUCHULUKA KWAKE		
	Zokonza nokha/zophikidwa kunyumba	Zokonzedwa kale	Takeaway/restaurant	zophatikiza	kutchito/kusukulu	zina	Chigawo chamadzulo	Chigawo cha mamawa	Zochuluka motai?	zingati?	Zazikulu bwanji?

Approved by
College of Medicine
11-Apr-2022
(COMREC)
Research and Ethics Committee

Nambala ya wotenga nawo mbali………………..

Approved by
College of Medicine

11-Apr-2022

(COMREC)
Research and Ethics Committee

Nambala ya wotenga nawo mbali………………..

GAWO J: FOOTBALL EXPOSURE, INJURY, ILLNESS, AND MEDICATIONS USE SURVEILLANCE FORMS

Player's Baseline Information Form

Team name:

Player's code No.	Playing position	Age (years)	Stature (cm)	Body mass (kg)	Dominant leg (L, R, B)	Details of previous major injuries	Study specific variable	Study specific variable

Approved by
College of Medicine
11-Apr-2022
(COMREC)
Research and Ethics Committee

Nambala ya wotenga nawo mbali………………..

Exposure Report Form
(for the documentation of team exposures)

(Team) Player's name: ________________________________

Date	Match / Training	No. of players (fully participating in training)	Duration of training session (minutes)	Study specific variable	Study specific variable

Approved by
College of Medicine
11-Apr-2022
(COMREC)
Research and Ethics Committee

Nambala ya wotenga nawo mbali………………..

Exposure Report Form
(for the documentation of individual player's exposure)

(Teams) Player's name:______________________________________

**Date**							
**Training / Match**							
**Study specific variable**							
**Study specific variable**							
**Player name.**	Report the duration of training and match play for each player (minutes)						

Approved by
College of Medicine
11-Apr-2022
(COMREC)
Research and Ethics Committee

Nambala ya wotenga nawo mbali………………..

Injury Report Form

(Team) Player: ______________________ Date: ________________

1A Date of injury: ________________ **1B Date of return to full participation:** ________________________

2A Injured body part

☐ Head/face ☐ shoulder/clavicula ☐ hip/groin
☐ Neck/cervical spine ☐ upper arm ☐ thigh
☐ Sternum/ribs/upper back ☐ elbow ☐ knee
☐ Abdomen ☐ forearm ☐ lower leg/Achilles tendon
☐ Low back/sacrum/pelvis ☐ wrist ☐ ankle
☐ Hand/finger/thumb ☐ foot/toe

2B Injured body part

☐ Right ☐ left ☐ not applicable

3 Type of injury

☐ Concussion with or without Loss of consciousness ☐ lesion of meniscus or cartilage ☐ haematoma/contusion bruise
☐ Fracture ☐ muscle rupture/strain tear/cramps ☐ abrasion
☐ Other bone injury ☐ laceration
☐ Dislocation/subluxation ☐ tendon injury/rupture tendinosis/bursitis ☐ nerve injury
☐ Sprain/ligament injury ☐ dental injury
☐ Other injury (please specify):…………………………………………….

4 Diagnosis (text or Orchard code):……………………………….. **4b Severity** (absence in days) …………

5 Has the player had a **previous injury** of the same type at the same site (i.e. this injury is a recurrence)?

☐ no ☐ yes

If **YES,** specify date of player's return too full participation from previous injury:

6 Was the injury caused by **overuse** or **trauma?**

☐ Overuse ☐ trauma

7 When did the injury occur?

☐ Training ☐ match

8 Was the injury caused by **contact** or **collision?**

☐ No ☐ yes, with another player ☐ Yes, with the ball ☐ Yes, with other object (specify)

9 Did the referee indicate that the action leading to the injury was a **violation of the laws?**

☐ No ☐ yes, free kick/penalty ☐ yes, yellow card ☐ yes, red card

If **YES**, was the referee's sanction against ☐ injured player ☐ opponent

10a How was the injury managed on the field of play? **10b** How was the injury managed thereafter? (incl referrals)

Approved by
College of Medicine
11-Apr-2022
(COMREC)
Research and Ethics Committee

Nambala ya wotenga nawo mbali………………..

Illness/ailment record (adapted from Mkumbuzi et al., 2021)

Player's name	Ailment	Cause/aetiology	Treatment administered

Approved by
College of Medicine
11-Apr-2022
(COMREC)
Research and Ethics Committee

Nambala ya wotenga nawo mbali………………..

Medications use report form

Player’s name	Drug class	Date given	Dosage	Reason for prescription and dispensing

Approved by
College of Medicine
11-Apr-2022
(COMREC)
Research and Ethics Committee

Appendix C1: Support Letter from Football Association of Malawi (FAM).

Approved by
College of Medicine

11-Apr-2022

(COMREC)
Research and Ethics Committee
