# Supplementary material for: FIFA football nurse – A task sharing approach in sports and exercise medicine practice in grassroots women’s football in low- and middle- income settings. A study protocol for a cluster randomised controlled trial
Source: PLoS One. 2023 Sep 21;18(9):e0278428. doi: 10.1371/journal.pone.0278428 (PMC10513307; doi:10.1371/journal.pone.0278428)
Supplement: Attachment — Submitted filename: Response to reviewers.docx [file pone.0278428.s003.docx]

**AUTHORS’ RESPONSE**

| **Reviewer’s comment** | **Authors’ response** | **Action taken** |
| --- | --- | --- |
|  |  |  |
| Please ensure that your manuscript meets PLOS ONE's style requirements, including those for file naming. | This is noted | We have followed the PLoS ONE style requirements throughout the manuscript. |
| Thank you for stating the following financial disclosure: "The FIFA Football Nurse project is funded by the Women’s Football Development Department at Federation Internationale Football Association (FIFA). The funders were not involved in the design or write up of the protocol." Please state what role the funders took in the study. If the funders had no role, please state: "The funders had no role in study design, data collection and analysis, decision to publish, or preparation of the manuscript." If this statement is not correct you must amend it as needed.  Please include this amended Role of Funder statement in your cover letter. We will change the online submission form on your behalf. | Noted | The financial disclosure statement has been revised to:  “The FIFA Football Nurse project is funded by the Women’s Football Development Department at Federation Internationale Football Association (FIFA). The funders had no role in study design, data collection and analysis, decision to publish, or preparation of the manuscript.” |
| We note that you have stated that you will provide repository information for your data at acceptance. Should your manuscript be accepted for publication, we will hold it until you provide the relevant accession numbers or DOIs necessary to access your data. If you wish to make changes to your Data Availability statement, please describe these changes in your cover letter and we will update your Data Availability statement to reflect the information you provide. | The submitted manuscript is for a study protocol, which, if accepted, will not have any associated data at the time of acceptance/publication. | N/A |
| Thank you for stating the following in the Competing Interests section:  "We have read the journal's policy and the authors of this manuscript have the following competing interests: AM is Director of the Medical Department at FIFA. NSM is a technical consultant for FIFA’s Women’s Football Department." Please confirm that this does not alter your adherence to all PLOS ONE policies on sharing data and materials, by  including the following statement: "This does not alter our adherence to PLOS ONE policies on sharing data and materials.” (as detailed online in our guide for authors <http://journals.plos.org/plosone/s/competing.interests>).  If there are restrictions on sharing of data and/or materials, please state these. Please note that we cannot proceed with consideration of your article until this information has been declared.  Please include your updated Competing Interests statement in your cover letter. We will change the online submission form on your behalf |  | The competing interest declaration has been revised to:  “AM is Director of the Medical Department at FIFA; NSM is a technical consultant for FIFA’s Women’s Football Department. This does not alter our adherence to PLOS ONE policies on sharing data and materials.” |
| Your ethics statement should only appear in the Methods section of your manuscript. If your ethics statement is written in any section besides the Methods, please delete it from any other section. | Noted | The ethics statement was deleted elsewhere in the manuscript and is now only in the methods sections. |
| Please include captions for your Supporting Information files at the end of your manuscript, and update any in-text citations to match accordingly. | Noted | The following captions were included and the in text citations have been updated:  Figure 1. Schedule of enrolment and assessments for the FIFA Football Nurse protocol.  **Figure 2:** Proposed referral pathways for players with injuries or illnesses during the FIFA Football Nurse intervention.  **Figure 3:** FIFA Football Nurse recruitment strategy for participating teams in the Malawi Women’s Super League  **Supplementary materials**   1. Proof of funding for the study 2. Ethics committee approved protocol version |
| We note that the original protocol file you uploaded contains a confidentiality notice indicating that the protocol may not be shared publicly or be published. Please note, however, that the PLOS Editorial Policy requires that the original protocol be published alongside your manuscript in the event of acceptance. Please note that should your paper be accepted, all content including the protocol will be published under the Creative Commons Attribution (CC BY) 4.0 license, which means that it will be freely available online, and any third party is permitted to access, download, copy, distribute, and use these materials in any way, even commercially, with proper attribution.  Therefore, we ask that you please seek permission from the study sponsor or body imposing the restriction on sharing this document to publish this protocol under CC BY 4.0 if your work is accepted. We kindly ask that you upload a formal statement signed by an institutional representative clarifying whether you will be able to comply with this policy.  Additionally, please upload a clean copy of the protocol with the confidentiality notice (and any copyrighted institutional logos or signatures) removed. | We, the authors, and the funders give consent for the protocol to be publicly share should our manuscript be accepted. | We have attached a formal statement from the funders clarifying that we will be able to comply with this policy. |
| **EDITOR’S COMMENTS** | | |
| The protocol proposes to randomise 12 football teams to receive an intervention and 12 to receive a control. The teams are made up of individuals and therefore this should be described as a Custer randomised controlled trial. | Noted | In all applicable instances, the study is referred to as a cluster randomised trial throughout the manuscript. |
| The protocol should be guided by the "Consort 2010 statement: extension to cluster randomised trials". | We agree with the editor on the need to use the CONSORT 2010 statement for cluster randomised trials | In all applicable instances, we used the statement to guide our reporting. Additionally, the following statement was added to the ‘Materials and Methods’ section:  ‘This study will be a cluster randomised control trial, whose reporting will follow the Consolidated Standards of Reporting Trials (CONSORT) 2010 statement: extension to cluster randomised trials.” |
| The authors stated 6 objectives. But it is recommended to identify one as the primary objective and all others as secondary. | We agree with the reviewer on the need to identify a primary objective. | The objectives have been rephrased as:  *Primary objective*   - Compare sports medicine practices; injury prevention behaviours; injury risk parameters; incidence and prevalence of injuries and illnesses in teams with and without a Football Nurse during one competitive season in Malawi’s Women’s football league.   *Secondary objectives*   - Determine current sports medicine practices in women’s football teams in Malawi’s Women’s Football League. - Determine baseline knowledge, attitudes, and behaviours of football stakeholders (players, coaches, team management) in women’s football towards injury prevention programs. - Identify football injury risk factors among women’s football players in Malawi’s Women’s Football league. - Determine the incidence and prevalence of injuries, illnesses, and medication use during one competitive season in Malawi’s Women’s football league. - Determine treatment and referral protocols of injuries and illnesses during one competitive season in Malawi’s Women’s football league. - To determine the financial feasibility of the FIFA Football Nurse intervention in grassroots women’s football in Malawi |
| I recommend that the Objective 6 should be rephrased as the primary objective, which must have hypothesis to operationalise the objective by stating the primary outcome and its measurement scale. | We agree with the reviewer on the need to identify objective 6 as a primary objective. | The primary objective is now:  Compare sports medicine practices; injury prevention behaviours; injury risk parameters; incidence and prevalence of injuries and illnesses in teams with and without a Football Nurse during one competitive season in Malawi’s Women’s football league. |
| The current protocol has too many outcomes:  1. Increased knowledge of and practice of injury prevention strategies.  2. Reduced intrinsic and extrinsic injury risk  3. Lower incidence and prevalence of injuries, illnesses, and medication use  4. Increased referral of injuries to other healthcare providers  5. Increased completion of injury rehabilitation protocols.  I recommend for the authors to identify one primary outcome and use the rest as secondary and power the study on  the primary outcome noting that this is a cluster RCT. If the authors decided to keep the 5 outcomes, this has  implication on the design of the study including sample size calculation and analysis to adjust for multiple primary  outcomes and indicate what a success looks like (effect on at least one outcome or effect on all outcomes????). | We agree with the reviewer on the need to identify one outcome as a primary outcome and to have the other ones as secondary outcomes. | The section has been revised to,  “We hypothesise that, when comparing this intervention at a cluster level, at the end of the competitive season, compared to women’s football teams without a Football Nurse, women’s football teams with a Football Nurse will have higher scores for SEM team practice with increased knowledge and practice of injury prevention strategies.” |
| Revise the statistical analysis plan to reflect the guidance I have provided here. Use statistical methods appropriate  for analysing cluster randomised trials. | We agree with the editor on the need for appropriate statistical analyses for cluster randomised trials | And the data analyses section has been revised to:  Data will be analysed using the IBM Statistical Package for Social Sciences (SPSS) version 21. Comparability of participants in clusters in both arms will be assessed for potential confounding factors; namely, age, physical/anthropometric characteristics, playing experience, and playing position. Due to the relatively small number of clusters, to have relative robustness in the non-independent data, the analyses will be based on cluster-level summary measures *(Chibanda et al., 2015; Campbell et al., 2000).* Descriptive statistics using mean and standard deviation (SD) will be used to characterise continuous demographic variables and categorical variables will be presented as frequencies and percentages [n(%)]. Injury rates and corresponding 95% CIs will be calculated for all players as the number of injuries reported per 1000 hours of football exposure. Further analysis will include the severity, incidence and type of injury, chronicity, mechanism, and timing of injury.  For the primary outcome (team medical practice), the mean score per cluster will be calculated at cluster and arm level with mean and standard deviation (SD) and associated 95% CI estimated per arm using the standard t-test if the size of the clusters does not vary widely, otherwise the weighted t-test will be used. Multiple linear regression: namely, 2-way ANOVA of the mean score on arm and clusters will be used to estimate the difference in team medical practice and 95% CI associated with the FIFA Football Nurse intervention. For categorical variables, a Chi- square test and either independent t-tests or Mann-Whitney U tests, for continuous variables, will be performed to compare both groups at the start of the trial. For binary outcomes, the risk for each cluster will be calculated, and shown by strata and arm. The mean and SD of the log risk will be used to estimate the geometric mean and associated 95% CI for each arm of the study. Linear regression of the mean risk on strata and arm will be used to estimate the risk difference and 95% CI. The approximate variance for the mean risks will be obtained based on the residual mean square from a 2-way ANOVA on arm and cluster. A 95% CI for this will be calculated from the variance using a weighted t-test. Pre-defined sensitivity analyses include adjustment for baseline team medical practice and other confounding factors with imbalance (e.g., age, physical/anthropometric characteristics).  For the adherence data, descriptive statistics will be used to determine in which areas the football nurse programme is best and worst implemented. Data from the semi-structured interviews will be transcribed verbatim and thematic analyses will be used to group the common themes. Once this is completed, the experiences of women’s football stakeholders, and the barriers and facilitators towards the football nurse task sharing approach will be described using frequency analyses. All statistical tests will be two - sided and p values of <0.05 will be considered statistically significant. |
| Revise the title to include cluster randomised trial. | Noted | The new title is:  **FIFA Football Nurse – A task sharing approach in Sports and Exercise Medicine practice in grassroots women’s football in low- and middle- income settings. A study protocol for a cluster randomised controlled trial** |
| **REVIEWER 1** | | |
| *Does the manuscript provide a valid rationale for the proposed study, with clearly identified and justified research*  *questions?*  The research question outlined is expected to address a valid academic problem or topic and contribute to the base of knowledge in the field.  Reviewer #1: Yes |  |  |
| *Is the protocol technically sound and planned in a manner that will lead to a meaningful outcome and allow testing*  *the stated hypotheses?*  The manuscript should describe the methods in sufficient detail to prevent undisclosed flexibility in the experimental procedure or analysis pipeline, including sufficient outcome, neutral conditions (e.g. necessary controls, absence of floor or ceiling effects) to test the proposed hypotheses and a statistical power analysis where applicable. As there may be aspects of the methodology and analysis which can only be refined once the work is undertaken, authors should outline potential assumptions and explicitly describe what aspects of the proposed analyses, if any, are exploratory.  Reviewer #1: Partly | We were not sure which elements of this point we had partly addressed or neglected to do so. We have revised the methods and data analyses sections and we hope that we have now managed to fully describe the protocol. | In all applicable instances, we have made changes to the methods and data analyses sections and throughout the manuscript. |
| *Is the methodology feasible and described in sufficient detail to allow the work to be replicable?*  Descriptions of methods and materials in the protocol should be reported in sufficient detail for another researcher to reproduce all experiments and analyses. The protocol should describe the appropriate controls, sample size calculations, and replication needed to ensure that the data are robust and reproducible.  Reviewer #1: No | We were not sure which elements of this point we didn’t describe appropriately in a robust and reproducible manner. We have revised the methods and data analyses sections and we hope that we have now managed to fully describe the protocol in a robust and reproducible manner. | In all applicable instances, we have made changes to the methods and data analyses sections and throughout the manuscript. |
| *Have the authors described where all data underlying the findings will be made available when the study is complete?*  The PLOS Data policy requires authors to make all data underlying the findings described in their manuscript fully  available without restriction, with rare exception, at the time of publication. The data should be provided as part of the manuscript or its supporting information or deposited to a public repository. For example, in addition to summary statistics, the data points behind means, medians and variance measures should be available. If there are restrictions on publicly sharing data—e.g., participant privacy or use of data from a third party—those must be specified.  Reviewer #1: No | The submitted manuscript is for a study protocol, which, if accepted, will not have any associated data at the time of acceptance/publication. | n/a |
| *Is the manuscript presented in an intelligible fashion and written in standard English?*  PLOS ONE does not copyedit accepted manuscripts, so the language in submitted articles must be clear, correct, and unambiguous. Any typographical or grammatical errors should be corrected at revision, so please note any specific errors here.  Reviewer #1: Yes | n/a | N/A |
| In this study protocol, a two-arm cluster randomized control trial is being proposed to evaluate the effectiveness of implementing the intervention of Football Nurses for Women’s Football League in Malawi. The primary aim is to train FIFA Football Nurses. The secondary aim is to evaluate the effect of trained nurses on sports injury related outcomes compared to controls. The feasibility of training nurses will also be evaluated. | n/a | n/a |
| The specific objectives on pages 16 and 17 do not correspond to the objectives identified in the abstract. | We thank the reviewer for noting this discrepancy in the stated objectives. | We have revised the objectives in the abstract to match the specific objectives in the main text.  “The *primary objective of this study will be to c*ompare sports medicine practices; injury prevention behaviours; injury risk parameters; incidence and prevalence of injuries and illnesses in teams with and without a Football Nurse during one competitive season in Malawi’s Women’s football league.” |
| Additionally, the specific objectives fail to mention cost and sustainability. | We thank the reviewer for nothing this oversight. | We have included financial feasibility as a secondary objective.  “To determine the financial feasibility of the FIFA Football Nurse intervention in grassroots women’s football in Malawi.” |
| State that the study will be a cluster randomized controlled study. | Noted | In all applicable instances, the study is referred to as a cluster randomised trial throughout the manuscript. |
| Page 20: Ratio is misspelled. | Noted | The spelling has been corrected |
| Sample size section: To improve clarity, replace “attached” with “assigned.” | Noted | The statement has been revised to “Twelve (12) nurses will be recruited and one each will be assigned to the intervention teams.” |
| Sample size justification: Include comprehensive sample size/power justification by stating the statistical testing method and the power of the test. | We have included a more detailed sample size justification. | The sample size section has been revised to:  Twelve (12) nurses will be recruited and one each will be assigned to the intervention teams. Six (6) physiotherapists and/or doctors based in the district hospitals where the teams are located will also be recruited to each supervise the Football Nurses from two teams. The sample size calculation considers the intra-cluster correlation coefficient, the number of participants involved in the study, the expected effect of the football nurse intervention, and the power of the study. The sample size was calculated using team sports medicine practice as an outcome measure of interest. Using the sample size calculation (*Sewry et al. 2016*):  $C=1+\frac{\left( \sum_{\alpha^{2}} .+\sum_{\beta} . \right)^{2}\left[ \frac{\pi_{0}\left( 1-\pi_{0} \right)}{n}+\frac{\pi_{1}\left( 1-\pi_{1} \right)}{n}+k^{2}\left( {\pi_{0}}^{2}+{\pi_{1}}^{2} \right) \right]}{\left( \pi_{0}-\pi_{1} \right)^{2}}$  Where $C$ is the number of clusters, $\sum_{\alpha^{2}} \left( 95\% \right)=1.96$, $\sum_{\beta} .\left( 90\% \right)=1.28$, $\pi_{0}=0.38$ is the mean sports medicine practice level for the control group, $\pi_{1}=0.57$is the mean sports medicine practice level for the intervention group, based on $50\%$ increase, $n=25$ is the number of participants per cluster, $k=0.2$ is the inter-cluster coefficient of variation. We would have 90% power to detect this increase if the coefficient of variation is smaller (k = 0.15), and 90% power to detect a larger effect size if k = 0.22. Therefore, to detect a statistically significant change in the team sports medicine practice at a $95\%$ confidence level, with 90% power, we would need 7 clusters (teams) in the control and intervention groups respectively, with a minimum of 25 participants in each arm; that is, a total of 175 participants in the control group and 175 participants in the intervention group.  Since we will use trials with a fixed number of equal sized clusters, the trial will be feasible provided:  $C>n_{I}\times k,$  where $n_{I}$ is the number of participants under individual randomisation, otherwise we will determine the maximum available power to detect the pre-specified difference, or the minimum detectable difference under the pre-specified value for the power. |
| Data Analysis section: State how categorical variables will be summarized. Typically, this includes frequencies and percentages. | We agree with the reviewer that categorical variables should be presented as frequencies and percentages. | The following has been included in the data analysis section.  “Categorical variables will be presented as frequencies and percentages [n(%)].” |
| Consider revising this statement because it is not accurate. “Should there be a statistically significant change at a specific time point, then a one-way ANOVA will be performed to determine the specific outcome that is different between the groups.” Consider the following: “Should there be a statistically significant overall time by group effect, step- down tests will be performed to determine at which time points the two groups differ.” | We agree with the reviewer to revise the statement. | The following revision has been made,  “Should there be a statistically significant overall time by group effect, step- down tests will be performed to determine at which time points the two groups differ” |
| To assist in the review process, add line numbering to the document. | We agree with the reviewer that line numbering would assist with reviewing the manuscript. | Line numbers were added to the manuscript. |
