# Supplementary material for: FIFA football nurse – A task sharing approach in sports and exercise medicine practice in grassroots women’s football in low- and middle- income settings. A study protocol for a cluster randomised controlled trial
Source: PLoS One. 2023 Sep 21;18(9):e0278428. doi: 10.1371/journal.pone.0278428 (PMC10513307; doi:10.1371/journal.pone.0278428)
Supplement: Attachment — Submitted filename: 1_A Response to reviewers .docx [file pone.0278428.s004.docx]

**AUTHORS’ RESPONSE**

| **Editor’s comments** | **Authors’ response** | **Action taken** |
| --- | --- | --- |
|  |  |  |
| There are several outcomes in the current primary objective including: sports medicine practices, injury prevention behaviours; injury risk parameters; incidence and prevalence of injuries and illnesses in teams. | We agree with the editor that the primary objective contained several outcomes. | The primary objective has been rephrased to: To estimate the effect of football nurse intervention on team sports medicine practice during one competitive season in Malawi’s Women’s football league. |
| The sample size calculation was based on “sports medicine practices”, suggesting that sports medicine practices is the primary outcome of interest. Therefore, authors should revise the primary objective to read as follows:  Primary Objective:  To estimate the effect of football nurse intervention on sports medicine practices during one competitive season in Malawi’s Women’s football league.  Hypothesis: Compared to teams without a Football Nurse, the [mean??] sports medicine practices will be higher among teams with a Football Nurse during one competitive season in Malawi’s Women’s football league. | We thank the editor for noting this. | The primary objective and hypothesis have respectively been rephrased to:   - To estimate the effect of football nurse intervention on team sports medicine practice during one competitive season in Malawi’s Women’s football league. - We hypothesise that compared to teams without a Football Nurse, the mean sports medicine practice will be higher among teams with a Football Nurse during one competitive season in Malawi’s Women’s football league. |
| The authors defined “sports medicine practices” as a mean and yet they used sample size formular for proportion? Can the authors use appropriate sample size formular. | We thank the editor for noting this discrepancy in our calculation and write-up | Sports medicine practice has been consistently referred to as a mean, and the sample size formula now reflects this. The sample size calculation and design has been revised to:  The study aims to evaluate the impact of the Football nurse intervention on team sports medicine practice. Hence, the study will recruit twelve (12) nurses, with one assigned to each intervention team, and six (6) physiotherapists and/or doctors to supervise the Football Nurses from two teams each.  To determine the appropriate sample size, the study used the cluster randomized formula as in previous studies (Sewry et al., 2006). This formula considers the intra-cluster correlation coefficient, number of participants, expected effect size, and power of the study. The formula is as follows:  $C=1+{(Z_{\alpha/2}+Z_{\beta})}^{2}\frac{[\sigma_{0}^{2} +\sigma_{1}^{2}]\times[1+(m-1)k]}{m{(\mu_{0}-\mu_{1})}^{2}}$  Where C is the number of clusters required per treatment arm, $Z_{\alpha/2}$ and $Z_{\beta}$ are the critical values of the standard normal distribution for the desired confidence level and power, respectively, $m$ is the number of individuals per cluster and $k$ is the intracluster coefficient, $\mu_{1}$ and $\mu_{0}$ are the true means, and $\sigma_{1}$ and $\sigma_{0}$ are the standard deviations,  of the outcome variable in the presence and absence of the intervention, respectively.  The design effect of a CRT is related to the intracluster correlation coefficient as follows:  $DEFF=1+(m-1)k$  Assuming a $95\%$ confidence level ($Z_{\alpha/2}=1.96$), a power of $80\%$ ${( Z}_{\beta}=0.84$), an expected effect size of 0.19 (based on a $50\%$ increase in team sports medicine practice), a standard deviation of $0.30$, an intra-cluster correlation coefficient of $k=0.2$, and 25 participants per cluster, the study estimates that it will need 12 clusters (teams) in both the control and intervention groups. Therefore, a total of 600 participants will be recruited, with 300 participants in each group. In summary, the study will recruit twelve (12) nurses and six (6) physiotherapists/doctors to supervise the intervention teams. The study will need 12 clusters (teams) in both the control and intervention groups, with a total of 600 participants (300 in each group) to detect a statistically significant change in team sports medicine practice at a $95\%$ confidence level and $80\%$ power, using the cluster randomized formula (Sewry et al., 2016; Hayes, R. J., & Moulton, L. H., 2017, §7.2.4) |
| Authors should add the remaining components (minus sport medicine practice) of the current primary objective to the list of secondary objectives as:  Compare injury prevention behaviours; injury risk parameters; incidence and prevalence of injuries and illnesses in teams with and without a Football Nurse during one competitive season in Malawi’s Women’s football league. | We thank the editor for this suggestion | The secondary objectives have been revised to:   - Determine current sports medicine practices in women’s football teams in Malawi’s Women’s Football League. - Determine baseline knowledge, attitudes, and behaviours of football stakeholders (players, coaches, team management) in women’s football towards injury prevention programs in teams with and without a Football Nurse during one competitive season in Malawi’s Women’s football league. - Identify football injury risk factors among women’s football players in teams with and without a Football Nurse during one competitive season in Malawi’s Women’s football league. - Determine the incidence and prevalence of injuries, illnesses, and medication use in teams with and without a Football Nurse during one competitive season in Malawi’s Women’s football league. - Determine treatment and referral protocols of injuries and illnesses in teams with and without a Football Nurse during one competitive season in Malawi’s Women’s football league. - To determine the financial feasibility of the FIFA Football Nurse intervention in grassroots women’s football in Malawi. |
| The statistical analysis plan looks like a shopping list of statistical techniques as if they don’t know which techniques suits what? For each outcome (or group of outcomes measured on similar scale), the authors should indicate the technique to use. For example, the authors should decide on whether linear regression or ANOVA is appropriate for cluster level analysis of continuous outcomes; decide on “t-tests or Mann-Whitney U tests, for continuous variables”???? etc | We agree with the editor that we should be more specific with which tests will be used to analyse which variables. We have also consulted the recommended text for guidance. | The data analysis plan has been revised to:  Data will be analyzed using the IBM Statistical Package for Social Sciences (SPSS) version 21. The Shapiro-Wilk test for normality of the data will be conducted. Baseline comparability of participants for potential confounding factors such as age, length of football career, and baseline injury risk will be conducted using a chi-square test (for categorical variables) and either independent t-tests [normally distributed data] or Mann-Whitney U tests [non-normally distributed data] (for continuous variables).  Due to the relatively small number of clusters, data analyses will be based on summary level measures to account for the shortcomings of individual level regression methods. The primary variable of interest will be sports medicine practice. The mean (SD) sports medicine practice score will be calculated and presented by cluster and by arm. The mean of these mean scores and their associated 95% confidence intervals (CI) will then be presented for each arm. Linear regression of the mean sports medicine practice scores per cluster and per arm; two-way analysis of variance (ANOVA), will be used to explore the differences in sports medicine practice score and the 95% CI associated with the FIFA Football Nurse intervention by arm.  For the injury, illness, and medication use data, injury, illness, or medication use history will be recorded to determine the baseline injury, illness, or medication use risk. Injury, illness, and medication use rates and corresponding 95% CIs will be calculated for all players as the number of injuries/illnesses/medications reported per 1000 hours of football exposure (training and match). The severity of the injuries/illnesses, as scored by the number of days of missed training or matches, will also be calculated. Additional analysis will include, but not be limited to, the incidence and type of injury/illness/medication; chronic versus acute injuries/illnesses/medications; contact versus non-contact injuries and mechanism of injury (all per 1000 player hours). A Poisson regression will be used to compare differences in the injury, illness, or medication use rates between the control and intervention arms. To evaluate the impact of the FIFA Football nurse intervention on injury/illness/medication use outcomes, we will use logistic regression analysis to compare the proportion of players with injuries/illness/taking medications between the intervention and control arms, adjusting for potential confounding variables such as previous injury, age, playing experience, BMI, and playing conditions. We will also conduct subgroup analyses to determine whether the intervention has differential effects on different subgroups of players.  For the testing data such as injury risk (FMS, musculoskeletal screening), nutritional data (24h recall), and mental health assessment (SMHART-1), a mixed between-within-subjects ANOVA will compare the two arms over the whole intervention, then a post hoc Bonferroni test will be used to determine whether there are significant changes in the various scores and at what time point during the intervention these occur. Should there be a significant change in test scores, a one-way ANOVA will be performed at the specific time point to determine which specific test is different between arms. Additionally, a mixed effects logistic regression model will be used to examine the effects of arm assignment (intervention vs control) and time for categorical outcomes (e.g., sports injury prevention behaviours). To assess adherence and feasibility, descriptive statistics will be used to determine which aspects of the FIFA Football Nurse programme are best or worst implemented. Sensitivity analyses include adjustment for baseline team sports medicine practice and other potential confounding factors (e.g., previous injury, age, football experience, BMI, socioeconomic status). We will also include tests for effect-modification by relevant factors.  Additionally, data from the semi-structured interviews will be transcribed verbatim and a thematic analysis will be conducted using Braun and Clarke’s 6 step model (Findlay et al., 2020) to group common themes and describe the experiences of women’s football stakeholders, and the barriers and facilitators towards the football nurse task-sharing approach. Analysis will be approached inductively by tagging meaning units which will then listed as themes, compared, and clustered to form categories, then assigned as general dimensions. Inter-rater reliability checks will be conducted by a blinded researcher, who will code a subset of the manuscripts and determine congruence of resulting themes. If clustering effects are observed in the data, these will be accounted for using appropriate statistical methods such as multilevel or hierarchical models.  All statistical tests conducted in this trial will be two-sided and p-values of < 0.05 will be considered statistically significant. |
| The authors are encouraged to read “Cluster Randomised Trials by Richard J. Hayes and Lawrence H. Moulten” for insight into analysis of cluster RCT. | We thank the editor for this suggestion, we have consulted it and found it quite informative | n/a |
| The subtitle in line 294 should read “Control or Standard of Care” | Noted | The subtitle has been revised to:  **Control or Standard Care** |
| The subtitle in line 306 should read “Football nurse intervention” | Noted | The subtitle has been revised to:  **Football nurse intervention** |
| The authors should integrate the “RCT methodology” section into the “ Trial design” section to read logically. | We agree with the editor that integrating this into one section improves the flow of the methods. | The ‘Trial design’ section has been revised to: |
